# Interaction of anions with the surface of a coordination cage in aqueous solution probed by their effect on a cage-catalysed Kemp elimination†

**DOI:** 10.1039/d1sc04887b

**Published:** 2021-10-25

**Authors:** Michael D. Ludden, Christopher G. P. Taylor, Max B. Tipping, Jennifer S. Train, Nicholas H. Williams, Jack C. Dorrat, Kellie L. Tuck, Michael D. Ward

**Affiliations:** Department of Chemistry, University of Warwick Coventry CV4 7AL UK m.d.ward@warwick.ac.uk; Department of Chemistry, University of Sheffield Sheffield S3 7HF UK; School of Chemistry, Monash University Melbourne VIC3800 Australia

## Abstract

An octanuclear M_8_L_12_ coordination cage catalyses the Kemp elimination reaction of 5-nitro-1,2-benzisoxazole (NBI) with hydroxide to give 2-cyano-4-nitrophenolate (CNP) as the product. In contrast to the previously-reported very efficient catalysis of the Kemp elimination reaction of unsubstituted benzisoxazole, which involves the substrate binding inside the cage cavity, the catalysed reaction of NBI with hydroxide is slower and occurs at the external surface of the cage, even though NBI can bind inside the cage cavity. The rate of the catalysed reaction is sensitive to the presence of added anions, which bind to the 16+ cage surface, displacing the hydroxide ions from around the cage which are essential reaction partners in the Kemp elimination. Thus we can observe different binding affinities of anions to the surface of the cationic cage in aqueous solution by the extent to which they displace hydroxide and thereby inhibit the catalysed Kemp elimination and slow down the appearance of CNP. For anions with a −1 charge the observed affinity order for binding to the cage surface is consistent with their ease of desolvation and their ordering in the Hofmeister series. With anions that are significantly basic (fluoride, hydrogen carbonate, carboxylates) the accumulation of the anion around the cage surface accelerates the Kemp elimination compared to the background reaction with hydroxide, which we ascribe to the ability of these anions to participate directly in the Kemp elimination. This work provides valuable mechanistic insights into the role of the cage in co-locating the substrate and the anionic reaction partners in a cage-catalysed reaction.

## Abstract 2

A cage-catalysed Kemp elimination reaction of 5-nitro-1,2-benzisoxazole (NBI) with hydroxide to give 2-cyano-4-nitrophenolate (CNP) as the product is sensitive to binding of different types of anion to the cage surface.

## Graphical abstract

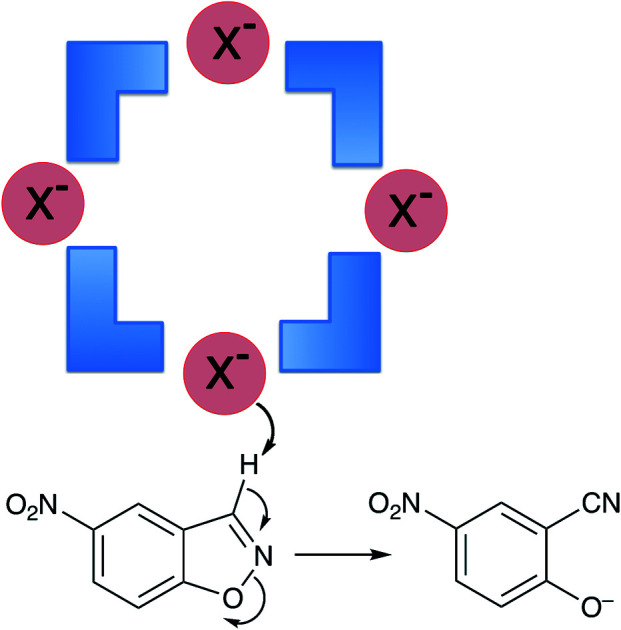

## Introduction

**Fig. 1:**
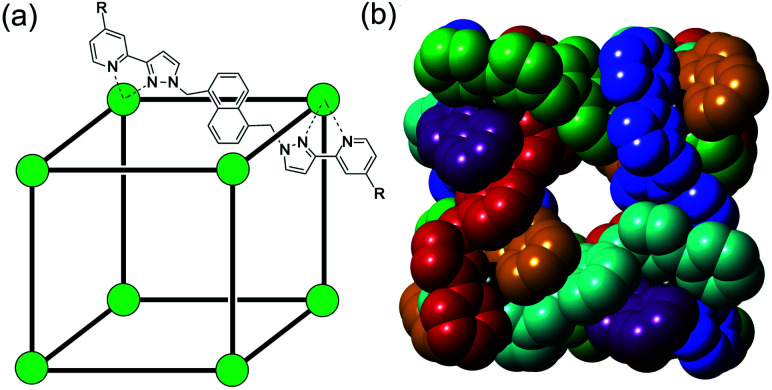
The host cage [Co_8_L_12_]^16+^, abbreviated as **H** (R = H) or **HW** (R = CH_2_OH). (a) A sketch emphasising the cubic array of Co(ii) ions and the disposition of one bridging ligand; (b) a space-filling view of the core (without the CH_2_OH substituents) showing each ligand coloured separately for clarity.

The ability of coordination cages to act as molecular containers, binding small-molecule guests inside their central cavity,^1–4^ is now very well established and has led to a wide range of functional behaviours including catalysis,^2^ sensing,^3^ and transport.^4^ As part of our work in this area using an octanuclear cubic M_8_L_12_ cage (Fig. 1),^5^ we have demonstrated that the accumulation of counter-ions around the exterior surface of a cationic cage is just as important a phenomenon as the binding of neutral guest molecules inside the cavity.^6–10^ This is a conclusion that has also been reached by others who have demonstrated how interactions of metal/ligand cage assemblies with counter-ions at the exterior surface can play fundamentally important roles in controlling structure and speciation behaviour of cage assemblies, as well as their ability to bind guests in the central cavity.^11–13^ Notably, Lusby and co-workers recently demonstrated how the positive charge associated with the surface of a cage host based on poorly-coordinating anions plays a crucial role in cage-catalysed reactions by stabilising anions, enhancing the acidity of reacting partners by several p*K*_a_ units;^12^ and a while ago Raymond and co-workers demonstrated the same effect in the opposite sense, with a highly negatively charged cage strongly stabilising (by 4 p*K*_a_ units) protonation of a bound guest, leading to the possibility of acid-based catalysis even under weakly basic conditions.^13^ Such charge-based effects are, clearly, equally as important as the more obvious host/guest effects based on cavity size and shape when considering the properties of cage-based systems.

The two types of guest binding that we have observed with our cage system in water – cavity-based binding of neutral hydrophobic organic molecules, and the binding of anions in the surface portals on the faces of the cage – have different origins with the former being substantially driven by the hydrophobic effect, and the latter by an electrostatic ion-pairing effect.^5^ The result is that the host cage co-locates the two guest types, bringing neutral organic species and anions into close proximity, which is the basis of catalysis of a range of reactions.^5–9^ The best example of cage-based catalysis we have demonstrated is the Kemp elimination: reaction of cavity-bound benzisoxazole with the shell of closely-adjacent hydroxide ions that accumulated around the cage surface, effectively giving a high local pH even when the bulk pH was modest. This effect resulted in a rate acceleration of up to 2 × 10^5^ fold compared to the uncatalysed reaction under the same conditions. The high catalytic turnover (>100 cycles with no loss of activity) arises because the reaction product, the 2-cyanophenolate anion, is sufficiently hydrophilic to exit the cavity and preferentially reside in the external aqueous phase, thereby ensuring no loss of activity due to the product blocking the cage cavity.^6^

We subsequently showed that the 2-cyanophenolate anion could itself accumulate around the cage surface and act as the base to deprotonate a cavity-bound benzisoxazole molecule in an autocatalytic cycle.^7^ It was apparent from this work and other control experiments that the tendency of anions to accumulate around the M_8_L_12_ cage surface (which has a 16+ charge) is not just driven by electrostatic factors but has a strong hydrophobicity component, with anions that are relatively hydrophobic and weakly hydrated having a higher affinity for the cage surface compared to more hydrophilic and strongly hydrated anions.^6,7^ We were able to exploit this recently in development of a fluorescence-based displacement assay to evaluate the ability of different anions to bind to the cage surface.^10^ Fluorescein dianions at pH ≈ 8 are relatively hydrophobic because of their large aromatic surface area and bind strongly to the M_8_L_12_ cage surface in aqueous solution, completely quenching the fluorescein's fluorescence. Titrations with different analyte ions displaced the fluorescein units from the cage surface, restoring their fluorescence, to varying extents according to the binding affinity of the analyte anion: using this method we could generate an affinity order of different anions for the M_8_L_12_ cage surface.^10^

**Scheme 1:**
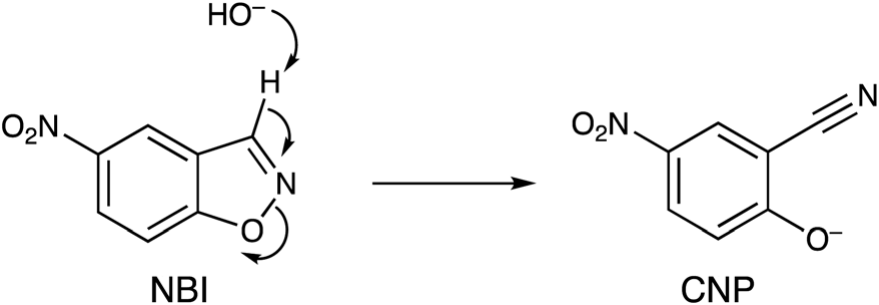
Kemp elimination reaction of 5-nitro-1,2-benzisoxazole (NBI) to generate 2-cyano-4-nitrophenolate (CNP).

In this paper we show how the differing binding abilities of different anions to the M_8_L_12_ cage surface can be used to modulate the catalysis of a Kemp elimination reaction using the substrate 5-nitro-1,2-benzisoxazole (NBI) (Scheme 1).^14^ This is a more tractable substrate to use for this cage-catalysed reaction than the original example (unsubstituted 1,2-benzisoxazole) as the reaction product 2-cyano-4-nitrophenolate (hereafter abbreviated CNP) has a strong absorbance at around 400 nm which can be conveniently monitored using UV/Vis spectroscopy rather than requiring ^1^H NMR spectroscopy, allowing large numbers of experiments to be run quickly and cheaply in parallel in a plate reader. The reaction itself, it should be emphasised, is not of major significance in terms of the importance of the product generated. However it provides a convenient way to monitor the effects of anion accumulation around the cage surface on cage-catalysed reactivity,^2^ and is equally relevant to control of catalysis in other ‘nanoreactors’ such as micelles and vesicles where catalysis can occur on the same basis – *viz.* by co-locating hydrophobic (in the cavity) and anionic (at the surface) reaction partners.^14*a*,15^

We found – somewhat to our surprise – that the cage-catalysed Kemp elimination of NBI occurs around the exterior surface of the cage rather than inside the cavity, as we have observed with some other substrates,^8,9^ despite the fact that NBI can occupy the cavity (as a crystal structure demonstrates). However, this does not hinder our ability to demonstrate how different anions affect the cage-based catalysis by displacing hydroxide ions (one of the reaction partners) from around the cage surface. This nicely illustrates the control that can be achieved in co-locating both neutral hydrophobic species and anions around the cage surface, a key component in developing further examples of cage-based catalysis.

## Results and discussion

### Guest binding and the structure of the cage/NBI assembly

**Fig. 2:**
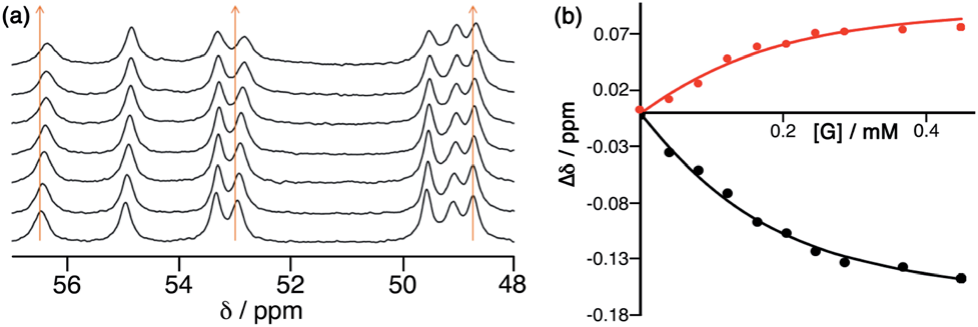
^1^H NMR titration of NBI into an aqueous solution of **H**·Cl_16_, showing (a) the small shifts in some signals as guest binding occurs in the cage cavity, and (b) fitting of some of these gradual shifts with added NBI to a 1 : 1 binding isotherm, giving *K* = 2 × 10^4^ M^−1^ (see main text).

The Co_8_L_12_ octanuclear cubic cage system used in this work (denoted **H** or **Hw**, according to the absence or presence of water-solubilising hydroxymethyl groups on the exterior surface, and usually prepared as the tetrafluoroborate salt), which has formed the basis of several studies on cage-based catalysis,^5–9^ is shown in Fig. 1. The use of NBI for further studies of the cage-catalysed Kemp elimination reaction, as mentioned above, is pragmatically driven by the ease with which multiple datasets can be measured using a UV/Vis plate reader given the strong visible absorbance of the CNP product anion. Our first priority was to investigate its binding in the cavity of the Co_8_L_12_ cage both in solution and the solid state. A ^1^H NMR titration in which aliquots of NBI were added to a solution of [Co_8_L_12_]Cl_16_ (**H**·Cl_16_) in water^7^ showed a small but steady shift of several of the (paramagnetically-shifted) NMR signals, indicative of guest binding being in fast exchange on the NMR timescale; fitting the data to a 1 : 1 binding isotherm afforded a *K* value of 2 × 10^4^ M^−1^ (Fig. 2). This is higher than we observed with unsubstituted benzisoxazole (4 × 10^3^ M^−1^),^6^ though the counter-ion of the cage used in that experiment was different for solubility reasons which can have an effect on *K* values, so the two numbers are not directly comparable. In water the dominant contribution to guest binding is the hydrophobic effect,^16^ and it has been shown recently that nitro substituents – despite their locally dipolar nature – are not effectively hydrated and best described as ‘hydroneutral’.^17^ The increase in surface area of NBI compared to unsubstituted benzisoxazole will therefore increase the contact area with the hydrophobic interior cage surface and displace more water molecules from the cage cavity when it binds, so a higher binding constant for NBI binding compared to benzisoxazole can be rationalised.^16*c*^ It is clear that NBI binds in fast exchange inside the cubic cage cavity in water, and that under the dilute solution conditions 1 : 1 host : guest binding occurs, as we have commonly seen with many other guests.^5^

**Fig. 3:**
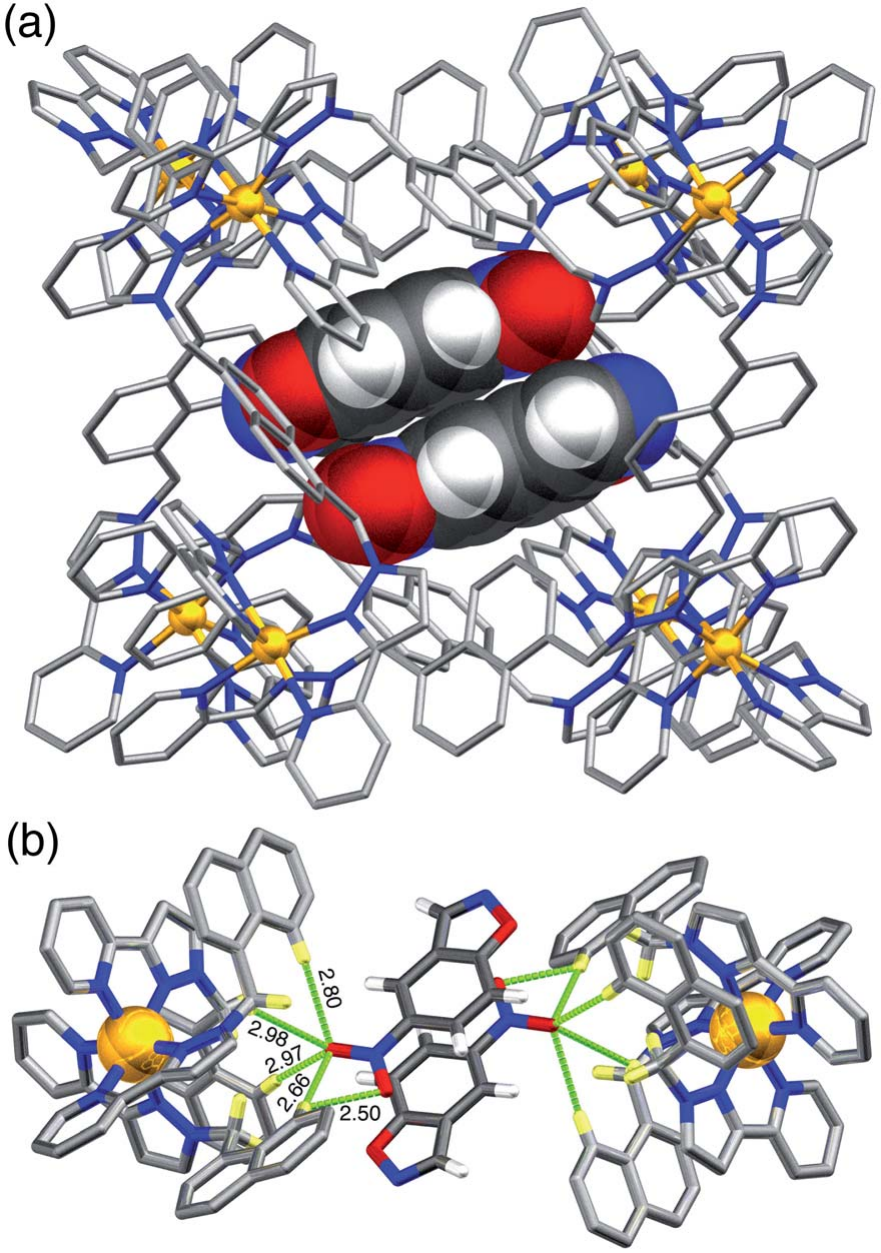
Crystal structure of the complex of host cage **H** containing a stacked pair of NBI guest molecules (lying either side of an inversion centre). (a) View of the whole complex with the host cage shown in wireframe and the two NBI guests, shown space-filling; (b) the network of CH⋯O hydrogen-bonding interactions (distances shown in Å) between the convergent set of inwardly-directed CH protons from the ligands which provide the H-bond donor site, and the O atoms of the nitro group on the guests which are the H-bond acceptors.

A crystal structure of the host cage complex with NBI bound in the cavity was determined (Fig. 3), with the sample prepared by the ‘crystalline sponge’ method^18^ that we have used before:^19^ a single crystal of [Co_8_L_12_](BF_4_)_16_ was immersed in a concentrated solution of NBI in MeOH for 24 hours, resulting in uptake of NBI guests into the cage cavity without loss of crystallinity. Structural analysis revealed that the cavity is occupied a stacked pair of symmetry-equivalent NBI guests (site occupancy 0.57 each) which lie across the crystallographic inversion centre at the centre of the cage molecule – an arrangement which we have seen with several other planar aromatic guests of comparable size.^19^ Based on a molecular volume for NBI of 144 Å^3^ and a cage cavity volume of 409 Å^3^ this leads to a cavity occupancy of 70% for the cage containing two NBI guests. This is higher than the value of *ca.* 55 ± 9% that is considered the optimal cavity occupancy in solution,^20^ but such high cavity occupancies are known in the solid state when a guest array is tightly packed because of *e.g.* π-stacking or hydrogen-bonding to the walls of the host,^19,21^ both of which occur here. We emphasise that this is not a reflection of what happens in dilute solution where – as the NMR titration showed – 1 : 1 binding dominates the speciation, because the crystalline sponge experiment is carried out under forcing, non-equilibrium conditions using a large excess of guest.^19^ In solution, even though a second guest could in principle bind, this will not be significant because – at the low concentrations used for spectroscopic measurements – *K*_2_ ≪ *K*_1_.

Each NBI molecule is oriented such that the nitro group is directed towards one of the two *fac* tris-chelate vertices, which lie at either end of the long diagonal of the cubic cage array, where there is an inwardly-directed set of CH protons from the ligand set which converge to make an H-bond donor site comparable in strength to a phenol.^22^ There are several CH⋯O interactions between these ligand protons and the electron-rich oxygen atoms of the nitro groups (distances in Å included in Fig. 3b). This type of H-bonding interaction between the electron-rich regions of guests and the H-bond donor site on the cage interior surface is a recurrent feature of these cage/guest structures,^5,19,22^ and contributes substantially to the strengths of guest binding in organic solvents.^23^ The stacked guests are exactly parallel to one another (because of the inversion centre) with their mean planes separated by a typical π-stacking distance of 3.32 Å. We note that this orientation of the guest in the cavity is different from what we observed with unsubstituted benzisoxazole, when it was the N and O atoms of the isoxazole ring that acted as the H-bond acceptors and are docked into the *fac* tris-chelate H-bond donor site,^6^ rather than (as here, with NBI) the nitro group: neutral organic nitro groups are known to be able to act as H-bond acceptors, albeit weakly, given the negative charge density on the O atoms.^24^ This difference of orientation of NBI compared to benzisoxazole in the cavity turns out to be significant (see later). As usual, the anions [tetrafluoroborate, arising from use of Co(BF_4_)_2_ in the cage synthesis] occupy the windows in the centre of each face, anchored by multiple CH⋯F hydrogen-bonds to the surrounding ligand array, such that six anions surround the cavity-bound guests.^5,7^

### Catalysis of the Kemp elimination reaction with NBI

**Fig. 4:**
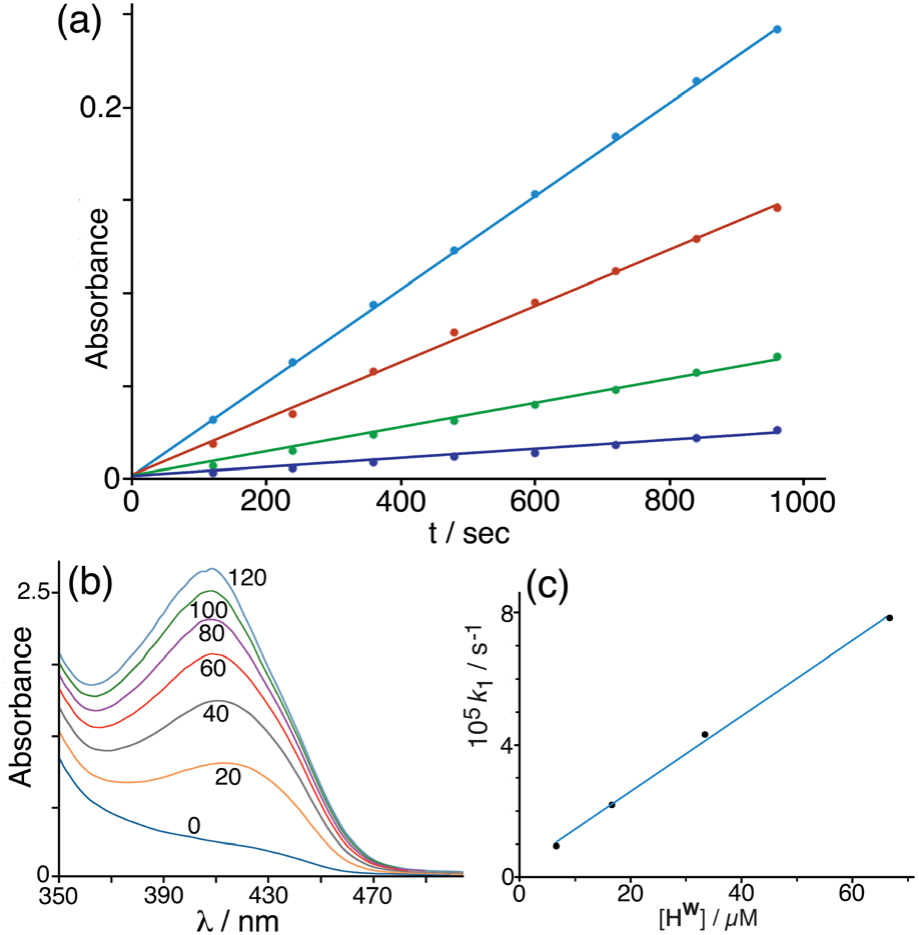
(a) Progress of the Kemp elimination reaction (background-corrected) using NBI as substrate at various catalyst concentrations (from bottom up: 6.7 μM; 16.7 μM; 33 μM; 67 μM): these show first order behaviour in NBI in the form of linear ln[NBI] *vs. t* plots. Reaction progress was monitored by absorbance at 408 nm. (b) Progress of a typical catalysed experiment (0.125 mM **Hw**) showing increasing absorbance from the CNP product with time (shown in minutes for each trace). (c) Observed reaction (initial) rate *vs.* catalyst concentration, confirming first order behaviour in catalyst **Hw.** Conditions: 298 K, pH 7; 0.25 mM NBI; varying concentrations of **Hw** as indicated. The slope of this line gives a value for the second-order reaction rate constant *k*_2_ of 1.18 M^−1^ s^−1^ which may be compared with the values given in Table 1 (entries a and p).

Given that NBI does bind inside the cage cavity in aqueous solution, we expected that the cage-catalysed Kemp elimination would occur by the same mechanism that we observed with unsubstituted nitrobenzisoxazole, with the cavity-bound substrate surrounded by a high local concentration of surface-bound hydroxide ions.^6^ This turned out not to be the case. Initial experiments did show clearly that the cage has a catalytic effect on the reaction (Fig. 4), with the background reaction of NBI with hydroxide ions in buffered solution at pH 7 being accelerated by addition of cage **Hw** to an extent that is linear with concentration of cage added (*i.e.* the catalysed reaction is first order in catalyst; see Fig. 4c).

Under the conditions used, the rate of the background reaction – conversion of NBI to CNP – had an observed first-order rate constant of 1.8 × 10^−5^ s^−1^; in the presence of 0.125 mM **Hw**, this increased to a total rate (background + catalysed) of 1.53 × 10^−4^ s^−1^ and the catalysed reaction was confirmed to be first-order in NBI based on analysis of initial rates during the first hour. Subtracting the background rate from the observed rate in the presence of **Hw** and dividing by catalyst concentration gives a second-order rate constant of *k*_2_ = 1.08 M^−1^ s^−1^ (Table 1, entry a) for the **Hw**-catalysed reaction at 298 K and pH 7. For comparison purposes this is *ca.* two orders of magnitude smaller than the equivalent figure for the catalysed reaction with benzisoxazole which occurs much more efficiently.^6^

**Table 1:** Second-order rate constants for **Hw**-catalysed Kemp-elimination reaction with NBI in the presence of different concentrations of added sodium salts^a^

Entry	Added anion (as Na^+^ salt)	Conc./mM	*k*_2_/M^−1^ s^−1^
a	None	—	1.08
b	F^−^	1.67	1.14
c	Cl^−^	1.67	0.81
d	Br^−^	1.67	0.29
e	NO_3_^−^	1.67	0.23
f	IO_3_^−^	1.67	0.89
g	HCO_3_^−^	1.67	1.51
h	SO_4_^2−^	1.67	0.85
i	F^−^	16.7	1.60
j	Cl^−^	16.7	0.34
k	Br^−^	16.7	0.12
l	NO_3_^−^	16.7	0.12
m	IO_3_^−^	16.7	0.84
n	HCO_3_^−^	16.7	Decomposes
o	SO_4_^2−^	16.7	0.73
p	None	—	1.24
q	F^−^	1.0	1.28
r	Formate	1.0	1.38
s	Acetate	1.0	1.54
t	HCO_3_^−^	1.0	2.15

a All experiments performed at 298 K and pH 7. Experiments a–o: 0.125 mM **Hw**; 0.2 mM NBI. Experiments p–t: 0.05 mM **Hw**; 0.1 mM NBI. All rate constants derived from initial-rate data during the first 2000 seconds. Estimated errors in *k*_2_, ±5%; all plate-reader measurements from which these rate constants are derived were performed in quadruplicate and averaged (see ESI).

A key observation indicates that, in this case, the catalysed reaction is not occurring in the cage cavity: blocking the cavity with an unreactive but strongly binding guest (cycloundecanone, CUD; *K* > 10^6^ M^−1^)^15^ does not significantly slow down the reaction. In our earlier work, with benzisoxazole as substrate, the very fast reaction was slowed in the presence of CUD to the background rate: *i.e.* the catalysis was completely inhibited when the cage cavity was blocked and the substrate could not bind.^6^ In contrast, with NBI as substrate, the reaction continues unchanged in the presence of CUD (see ESI†). We have observed before that some other substrates undergo cage-catalysed reactions with hydroxide ions at the exterior surface of the cage: the observations described above with NBI are consistent with this type catalysis occurring.^8,9^ The exterior surface is just as hydrophobic as the interior surface and so will allow some aggregation with hydrophobic species such as NBI. This association will be weaker than cavity-binding as the guest is not surrounded by the host so there will be less overlap of hydrophobic host and guest surfaces compared to cavity binding. Nonetheless, this brings the substrate into contact with the high local concentration of hydroxide ions that accumulate around the cage surface for electrostatic reasons. In addition, the reaction is occurring in a more favourable solvation environment for the product, in the exterior aqueous phase rather than the interior hydrophobic cavity (though this also solvates the hydroxide ions, reducing their activity). Catalysis of this type of elimination reaction is therefore not limited to the interior cavity of the cage, although our previous work with benzisoxazole showed that catalysis on the interior was far more efficient.^5,6^

**Fig. 5:**
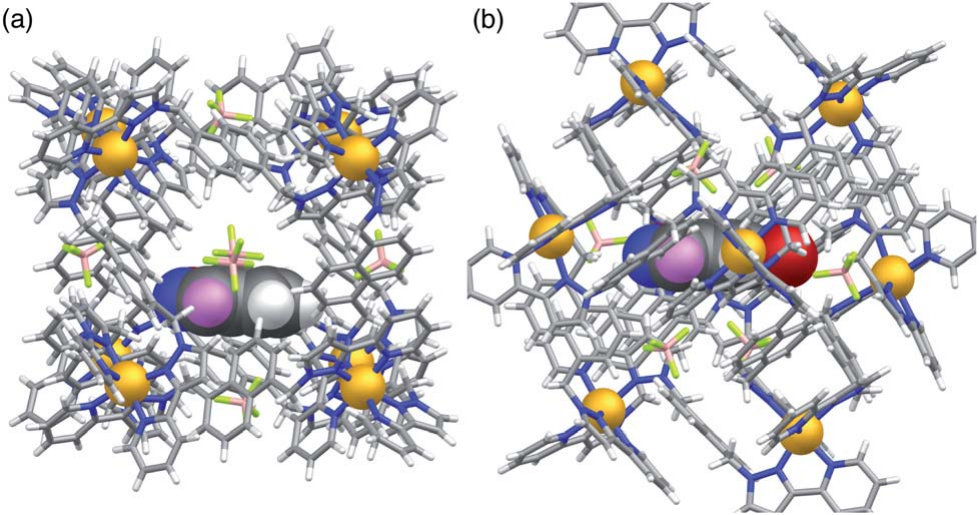
Molecular models of the host cage **H** containing (a) one molecule of benzisoxazole and (b) one molecule of NBI. The views are arranged to show the same edge-on orientation of the guest, looking down on to the C–H proton (coloured purple) that is the one extracted as the first step in the Kemp elimination. In (a) the C–H proton is clearly directed towards a portal and accessible to a surface-bound anion; in (b) the different orientation of the NBI guest in the cavity makes the C–H proton less accessible.

Given that NBI clearly does bind inside the cage cavity in solution, it is curious that cavity-bound catalysis does not work in the way that it does with unsubstituted benzisoxazole.^6^ Molecular modelling provides some insight to this. Molecular models of host cage **H** containing one molecule of either benzisoxazole or NBI as guest were calculated using the molecular docking program ‘GOLD’, by posing one molecule of the guest inside the cavity of a (rigid) host cage whose structure comes from crystallographic data (Fig. 5).^25^ With a single molecule of benzisoxazole as guest, in the energy-minimised structure the C–H proton that is removed during the Kemp elimination is directed towards a portal and is therefore accessible to a surface-bound anion (Fig. 5a). In contrast, with NBI as guest, its different orientation in the cavity – which was obtained as the minimum-energy structure from multiple different initial cage/NBI geometries, see ESI† – is associated with H-bonding of the nitro group to the cage interior surface (seen also in the crystal structure reported above, but with a stacked pair of guests). This means that the C–H proton is no longer directed towards a portal and – in this conformation of the adduct – is less accessible to a surface-bound anion (Fig. 5b). Thus, the different steric properties of the cage/guest complex could be significant here.

An additional possibility could be that, even if a cavity-bound NBI is accessible to a surface-bound hydroxide ion, the pathway to formation of the expanded, ring-opened product is inhibited in the cavity by the additional bulk of the nitro group. Similar loss of reactivity has been demonstrated for cage-bound P_4_, for example, arising from the fact that reaction of P_4_ with O_2_ results in initial formation of bulky intermediate species that cannot be accommodated in the confined space.^26^ The overall effect in our case is that catalysis does happen, but outside the cavity, and much more slowly than for the cavity-based reaction of unsubstituted benzisoxazole.^6^

### Interaction of the product phenolate anion with the cage surface

**Fig. 6:**
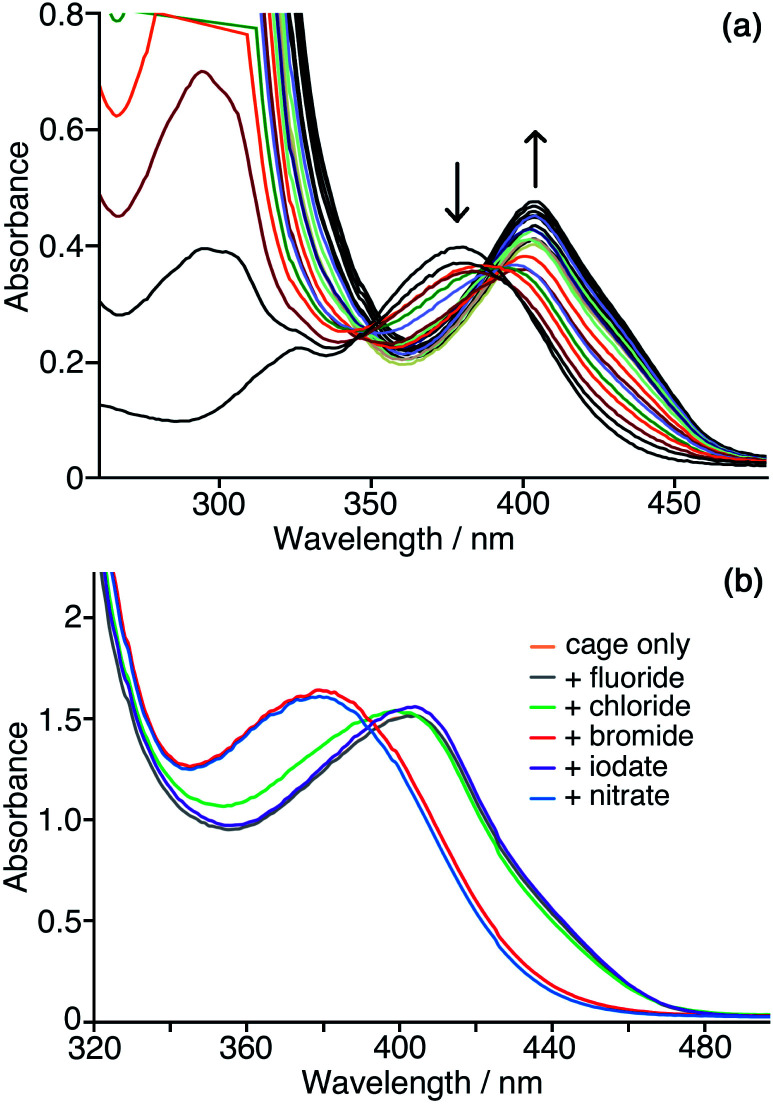
Interaction of the CNP anion with the cage surface. (a) Addition of small samples (2 μM concentration increments) of **H** to a solution of CNP (0.05 mM) in water, showing the red-shift in the CNP absorbance maximum as it binds to the cage surface; the red-shift of the maximum is complete after addition of *ca.* 0.3 equiv. of cage, thereafter only small changes in intensity occur. (b) Effects of addition different inorganic mono-anions (10 equiv.) to displace CNP from the surface of **Hw** in water. Conditions: 0.05 mM **Hw** and 0.1 mM CNP at pH 7. Note that the spectrum for ‘cage only’ (orange trace) is completely concealed under the spectrum recorded with added fluoride (purple trace), indicating the lack of ability of highly-solvated fluoride to displace CNP from the cage surface.

We need to highlight at this point an issue which complicates monitoring of the reaction progress using the absorbance of the CNP anion. During the catalysis studies described in this paper we noticed that the absorption maximum of the product anion CNP was red-shifted by the presence of **Hw**. In aqueous solution without cage present, *λ*_max_ for the lowest-energy charge-transfer absorption of CNP is 379 nm: but in the presence of **Hw** this is red-shifted to 405 nm, which we ascribe to interaction of the anion with the cage surface at the anion-binding sites in the windows.^6,7,10^ This red-shift happens in exactly the same way with both **Hw** and the unsubstituted but otherwise isostructural cage **H** (Fig. 6a) indicating that the hydroxymethyl pendant groups attached to **Hw** are not involved in this interaction: this is consistent with the windows at the face centres being involved, as we have seen crystallographically for many small inorganic anions.^5,27^ Significantly, this red-shift in *λ*_max_ for CNP is complete after addition of about 0.3 equivalents of either **H** or **Hw**, indicating that multiple (≈3) CNP anions can interact with a single cage molecule, consistent with surface rather than cavity binding. This is exactly consistent with the behaviour that we have seen with other large/soft aromatic anions such as fluorescein, where multiple anions can interact with the surface of a single **Hw** molecule depending on the concentrations of components.^10^

We can use this red-shift to put the binding affinity of the CNP anion for the cage surface into an affinity series with other anions. Fig. 6b shows the results of adding an excess of a range of different inorganic monoanions (halides, iodate, nitrate) to a solution of 0.05 mM **Hw** and 0.1 mM CNP at pH 7. Initially *λ*_max_ for CNP is at 405 nm, as the two equivalents of CNP are fully bound to **Hw**. Addition of 0.5 mM of fluoride or iodate (hydrophilic anions which bind weakly to the cage surface)^10^ did not change the *λ*_max_ value, indicating that these anions could not displace CNP even when present in excess. Addition of 0.5 mM of bromide or nitrate – more weakly hydrophilic anions which bind more strongly to the cage surface as they are easier to desolvate^10^ – in contrast results in *λ*_max_ for CNP shifting to 379 nm, which is the value for the free anion, indicating complete displacement. With addition of 10 equivalents of chloride (green line in Fig. 6b), an intermediate result is obtained, with a slight blue shift of *λ*_max_ to 399 nm and a change in curve shape with the emergence of increased absorbance between 340–380 nm associated with some free CNP: so we can see a slight amount of displacement of CNP from the surface of **Hw** but it is far from complete even in the presence of a large excess of chloride, indicating that the affinity order of these two is CNP > chloride. We know from our previous work that the affinity order of inorganic monoanions for the surface of **Hw** is nitrate > bromide > chloride > iodate > fluoride (following the Hofmeister series).^10^ We can now insert CNP into that sequence to give an affinity order of anions for the cage surface of nitrate > bromide > CNP > chloride > iodate > fluoride, with the hydrophobicity and ease of desolvation of CNP positioning it between chloride and bromide in the affinity order of anions.

This shift of *λ*_max_ for the CNP anion according to whether it is bound to the cage surface or free in aqueous solution – which depends on any competing anions present (Fig. 6) – affects the reaction monitoring because the proportion of bound *vs.* free CNP will change as the reaction proceeds, meaning that the absorbance at one fixed monitoring wavelength may not follow the Beer–Lambert law. We can partly compensate for this in two ways. Firstly, the absorbance for the catalysis reactions was monitored by recording UV/Vis spectra over the whole relevant range rather than just observing the absorbance at one wavelength, and the most appropriate monitoring wavelength was selected for each experiment calculation according to where *λ*_max_ was located. Secondly, rate constants discussed in this paper are based on measurements of initial rates during the early stages of the reaction when the curves fit well to simple first-order behaviour at a given catalyst concentration, and the absorption maximum had not significantly drifted because of a change in balance between free and cage-bound CNP.

### Effect of added anions on the cage-based catalysis

**Fig. 7:**
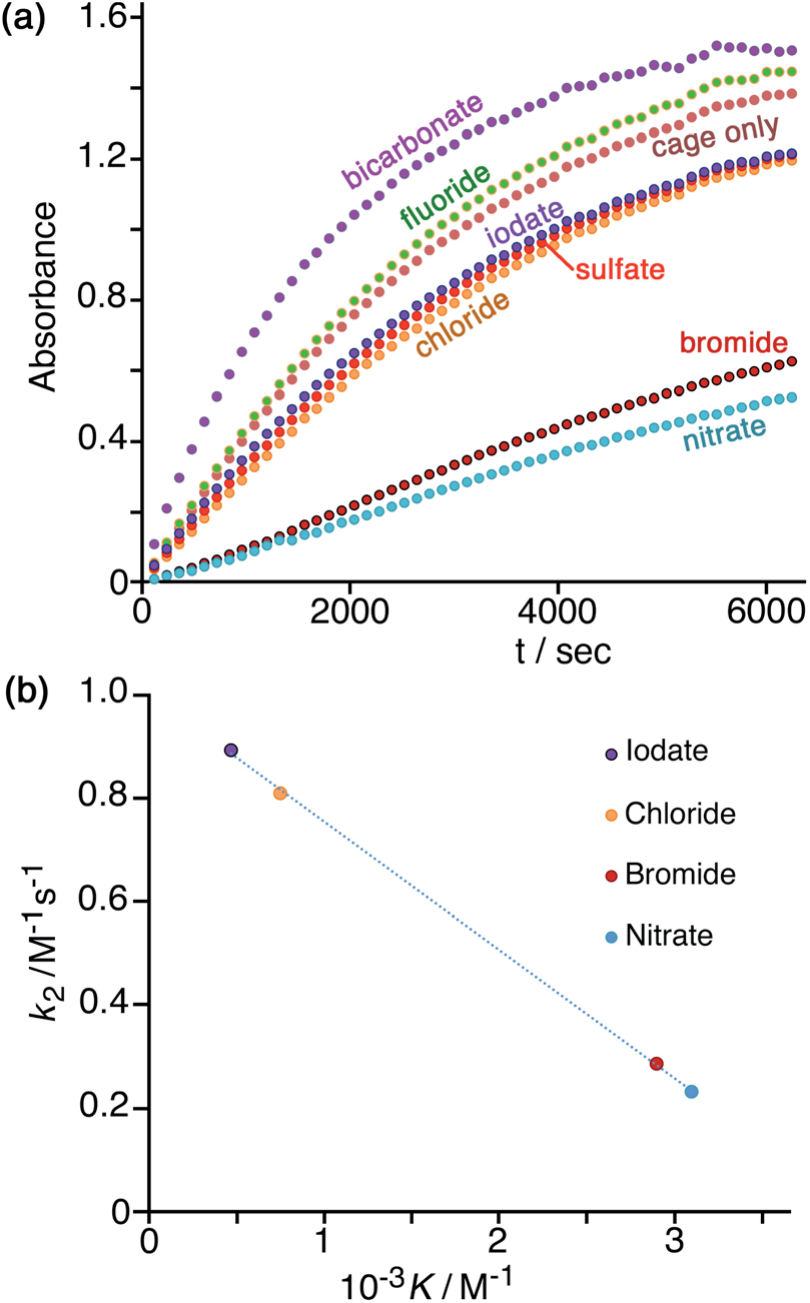
(a) Progress of cage-catalysed Kemp eliminations (background-subtracted) using NBI as substrate, monitoring formation of CNP by its absorbance in the 380–410 nm region (conditions: aqueous solution at pH 7 using 16.7 mM phosphate buffer; 0.125 mM **Hw**; 0.2 mM NBI; various different added anions as sodium salts, 1.67 mM. Shown are results from individual plate-reader experiments; four such repeats are averaged to give the *k*_2_ values in Table 1). (b) Correlation between reduction in second-order rate constant for catalysis (*k*_2_) in the presence of various anions (this work), based on analysis of initial rates, and the binding constant of that anion for a cage surface binding site (*K*, from ref. 10).

In Fig. 7a is shown the effect of adding a fixed concentration (1.67 mM, as the sodium salt) of a range of different anions to a catalysis reaction using 0.125 mM **Hw** and 0.2 mM NBI (buffered at pH 7). In the as-synthesised cage, 0.125 mM **Hw** (with its 16+ charge) is accompanied by 2 mM tetrafluoroborate, but hydrolysis of tetrafluoroborate following dissolution generates borate and fluoride^28^ which accordingly are also present as part of the baseline conditions, as is the ionic background from the 16.7 mM phosphate buffer: so the discussion of anion effects in this section is relative to the effects of this fixed background. The initial rates for the catalysed reactions in the presence of the different additional anions (with rates for the background reaction in the absence of catalysts subtracted) have been used to calculate the second-order rate constants in Table 1.

We can immediately see two contrasting effects, according to how basic the added anions are. Compared to the experiment with 0.125 mM **Hw** on its own, addition of non-basic anions reduces the reaction rate, with – for example – addition of 1.67 mM or 16.7 mM bromide causing a decrease in *k*_2_ by factors of *ca.* 3 or 9 respectively (Table 1, entries d and k). This is in line with what we have observed before:^7,10^ bromide has a higher affinity than does hydroxide for the cationic but hydrophobic cage surface, due to its smaller desolvation enthalpy.^29^ Bromide therefore preferentially accumulates around the cage, reducing the local concentration of hydroxide and slowing down the reaction. The order in which the added monoanions inhibit the Kemp elimination reaction is IO_3_^−^ < Cl^−^ < Br^−^ < NO_3_^−^, which is the same as the affinity order of these anions for the cage surface that we established recently using our recent fluorescence-displacement assay.^10^ In fact the correlation of the *k*_2_ value in the presence of these anions (measured in this work, Table 1) with *K* (the 1 : 1 binding constant with an anion-binding site in a cage face)^10^ is strikingly linear (*R*^2^ > 0.999; Fig. 7b): the extent of inhibition of the surface-catalysed Kemp elimination is directly related to the affinity of the anion for the cage surface. For chloride, bromide and nitrate this is the expected order based on the Hofmeister series.^30^ Iodate does not figure in some lists of the Hofmeister series,^30*a*^ but recently the iodate anion has been shown to be more strongly hydrated than many other oxyanions due to a highly polarised I(*δ*+)/O(*δ*−) charge distribution and therefore acts as a more powerful kosmotrope than chloride^31^ – exactly in agreement with what we observe.

The effect of sulfate, the only 2− anion of this series, on inhibiting the cage-catalysed Kemp elimination of NBI is however out of step with the high affinity of sulfate for the cage surface that we measured earlier.^10^ Although sulfate is expensive to desolvate,^29^ it nonetheless has a high affinity for the cage surface, possibly because its high desolvation enthalpy is offset by strong electrostatic attraction between 16+ cage and 2− anion: its 1 : 1 binding constant to a cage surface binding site is higher than that of bromide and nitrate.^10^ Its ability to inhibit the Kemp elimination of NBI by accumulating around the cage surface is therefore smaller than we expected (Table 1, entries h and o) on the basis of the 1 : 1 anion/cage binding constants. However there will be an additional electrostatic effect in play here: whilst one sulfate dianion binds strongly to **Hw**, its 2− charge limits the number of *additional* sulfates that will approach the cage. Indeed we observed before that such effects are significant, with the trianion of hydroxypyrene-tris(sulfonate) [HPTS]^3−^ forming a 6 : 1 HPTS : **Hw** complex under forcing conditions, but the tetra-anion [HPTS]^4−^ forming a neutral 4 : 1 HPTS : **Hw** complex at the same concentration.^10^ Thus, a small number of sulfate anions may indeed bind strongly to **Hw**, as the 1 : 1 *K* value suggests, but its ability to saturate the cage surface and exclude all hydroxide ions will be electrostatically inhibited, such that some hydroxide will still have access to the cage surface and facilitate the catalysed reaction with NBI, as we observe. It is notable that increasing the concentration of sulfate tenfold from 1.67 mM (Table 1, entry h) to 16.7 mM (Table 1, entry o) has very little additional effect on the inhibition.

**Fig. 8:**
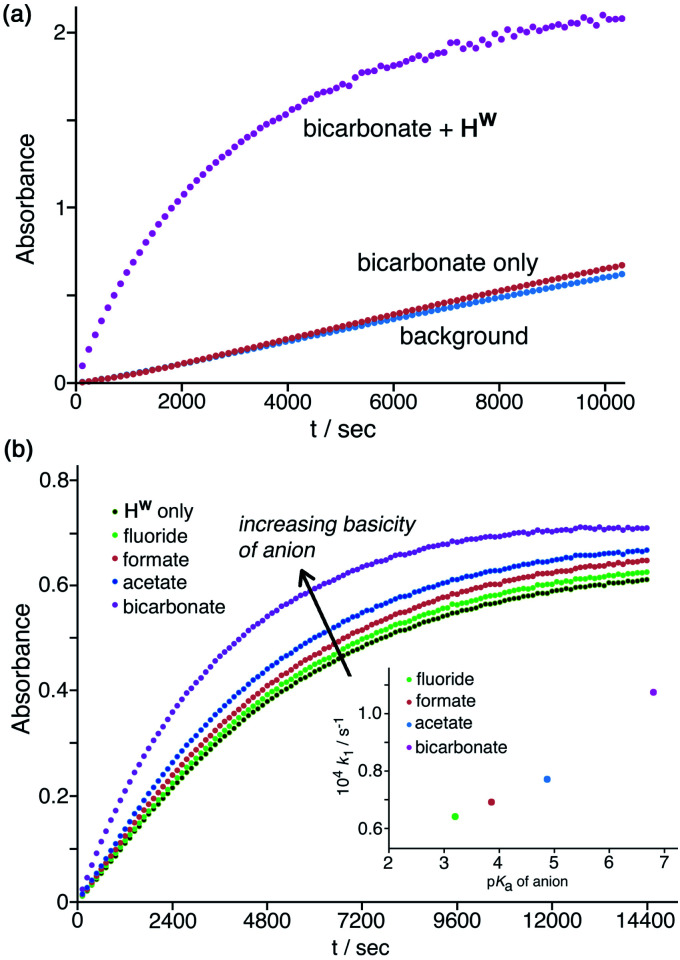
Effects of anion basicity on catalysis reaction rates. Shown are results from individual plate-reader experiments; four such repeats are averaged to give the *k*_2_ values in Table 1. (a) Experiment showing how added HCO_3_^−^ has no effect on the background reaction rate when **Hw** is not present; both **Hw** and HCO_3_^−^ need to be present to see the additional rate-accelerating effect. Conditions: 0.2 mM NBI; 0.125 mM **Hw**; pH 7 using 16.7 mM phosphate buffer. (b) Reaction progress curves (pH 7; 298 K; 0.05 mM **Hw**; 0.1 mM NBI) for the cage-catalysed Kemp elimination with NBI in the absence of any added anion, and then with added fluoride, formate, acetate or hydrogen carbonate as their sodium salts (1.0 mM) [inset: correlation of catalysed pseudo first-order reaction rates with p*K*_a_ of added anion].

Finally, these experiments are carried out in phosphate buffer, which is a mixture of mono- and dianionic inorganic phosphate, and so these specific electrostatic effects of more highly charged anions are already present in the background baseline activity. Hence, the specific electrostatic impact of dianionic sulfate (relative to the other anions) may already be present, and so the additional impact is much reduced under these conditions as sulfate substitutes for dianionic phosphate. In contrast to the effects of the above anions which all have an inhibitory effect, addition of a basic anion such as HCO_3_^−^*increases* the catalysed reaction rate, with *k*_2_ increasing slightly by a factor of *ca.* 1.5 (Table 1, entry g) by addition of 1.67 mM NaHCO_3_. Given that the solution is buffered at pH 7 this cannot be ascribed to a pH change on addition of NaHCO_3_, but it can plausibly be ascribed to the fact that the HCO_3_^−^ ions that will accumulate around the cage surface can act as bases in the Kemp elimination in a way that bromide, nitrate *etc.* do not. This result suggests that HCO_3_^−^ ions deprotonate NBI and initiate the Kemp elimination more rapidly than do hydroxide ions. This of course is inconsistent with the relative p*K*_a_ values of these anions: but it is consistent with the lower hydration enthalpy of HCO_3_^−^ resulting in a higher local concentration of it around the cage surface than the more strongly solvated HO^−^ ions can achieve.^29^ An experiment in the absence of cage (Fig. 8a) showed that this effect does not arise from the anions alone but requires the additional presence of **Hw** to bring the anions into close proximity with the NBI substrate, co-locating the two reaction partners. A 50% increase in reaction rate is small, likely because of (i) the high background concentration of other anions from the buffer and cage counter-ions, and (ii) the fact that the reaction is happening at the cage exterior surface where hydroxide ions can also access the substrate. However the fundamental difference between (i) the weakly accelerating behaviour of HCO_3_^−^, and (ii) the inhibitory nature of other non-basic anions, on the catalysis by **Hw** is very clear.

Similarly, addition of fluoride causes a small increase in reaction rate (Fig. 7a and 8b) which we ascribe to its weak basicity, as discussed above for HCO_3_^−^, though the effect is smaller (addition of 1.67 mM and then 16.7 mM fluoride results in increases in *k*_2_ by *ca.* 5% and then 50% respectively; Table 1, entries b and i). We investigated this effect of the basicity of the added anion further using the simple carboxylates acetate and formate in addition to fluoride and HCO_3_^−^, giving a p*K*_a_ range for the added anions ranging from 3.2 (fluoride), 3.8 (formate), 4.8 (acetate), and 6.4 (HCO_3_^−^) (Table 1, entries p–t). With the same concentration of added anion, all resulted in an increase in the catalysed reaction rate compared to the reaction rate in the presence of **Hw** alone, with a clear correlation between anion basicity and increase in reaction rate (Fig. 8b). The particularly substantial effect of HCO_3_^−^ may be because of the presence of traces of the more powerful base carbonate (p*K*_a_ 10.3). Although at pH 7 there should be very little of this present in bulk solution, the stabilising effect of the 16+ charge of the cage moves this p*K*_a_ value for surface-bound carbonate downwards,^10^ meaning that the rate-enhancing effect of added NaHCO_3_ may include a contribution from carbonate as well as from hydrogen carbonate. As mentioned above the absolute effects are small – a difference of >3 p*K*_a_ units between different anions results in just a factor of 2 difference in initial catalysed rates, as shown in Fig. 8b, and reasons for this have been suggested. However the trend is again clear and confirms the role of **Hw** in solution in co-locating the NBI substrate and the anion which acts as base to initiate the Kemp elimination reaction.

**Fig. 9:**
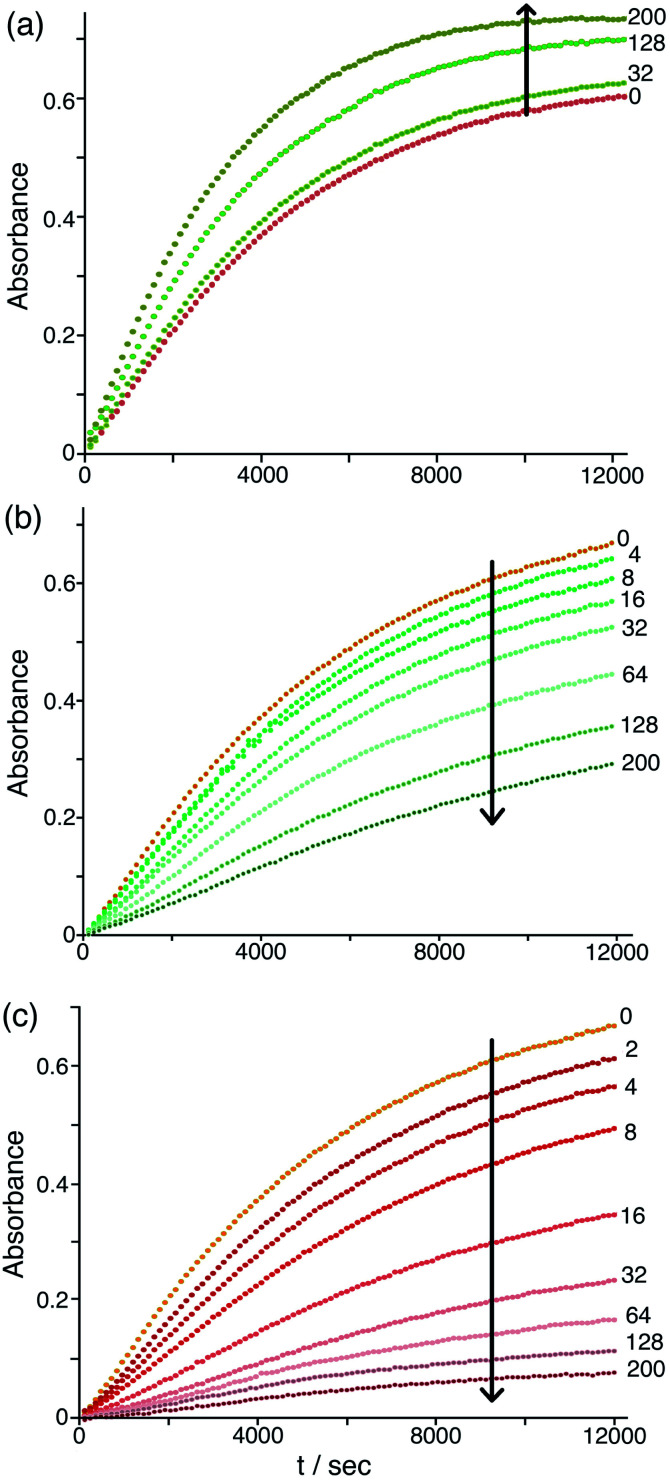
Effect of increasing concentrations of halide ions [(a) fluoride; (b) chloride; (c) bromide] on the progress of the **Hw**-catalysed Kemp elimination reaction of NBI (background subtracted), monitoring formation of CNP by its absorbance in the 380–410 nm region. Conditions: 16.7 mM pH 7 aqueous phosphate buffer; 0.05 mM **Hw**; 0.1 mM NBI; concentration of added halide, 0.05–10 mM. The numbers on the curves are numbers of equivalents of added halide ion per cage. Shown are results from individual plate-reader experiments; four such repeats are averaged to give the *k*_2_ values in Table 1.

The differing effects of the halides (chloride and bromide retarding the catalysis by displacing hydroxide from around the cage surface, but fluoride accelerating the catalysis because of its weakly basic nature) are clearly illustrated in Fig. 9 which shows the effects of adding up to 200 equivalents of these anions in small portions. On incremental additions of fluoride the reaction rate steadily increases until it has approximately doubled after 200 equivalents of fluoride are added. In contrast there are obvious reductions in reaction rate as more and more chloride or bromide are added, with bromide having a greater effect for the reasons discussed earlier. Overall these observations confirm the presence of two distinct effects on the catalysis associated with accumulation of anions around the surface of **Hw**: (i) the generally inhibiting effect on the Kemp elimination associated with displacement of hydroxide, to an extent depending on the binding affinity of the anions for the cage surface; but (ii) an accelerating effect in those cases where the new anion can itself act as base, as shown by the relationship between the magnitude of this effect and the anion basicity (Fig. 8b and 9a).

One final point to note in this section is that we can clearly see, in those cases when the reaction is most inhibited (notably in the presence of chloride, nitrate or bromide; *e.g.* in Fig. 7a and 9b, c), a slightly sigmoidal component to the reaction progress curve. This could be indicative of an autocatalytic^7,32^ mechanism becoming significant in which the product of the reaction – which here would be the CNP anion, itself a ‘soft’ (hence, weakly solvated) but weakly basic^33^ anion – acting as the base to deprotonate another equivalent of starting material and propagate the reaction, as we saw before with our cavity-bound Kemp elimination catalysis.^7^ In such situations the reaction accelerates as the product (the catalyst) accumulates, until substrate runs out, resulting in the characteristic sigmoidal shape for the reaction progress profile.^32^ However we can conclusively rule this out here. A simple test for autocatalysis is that adding a small amount of product to the start of the reaction should accelerate the reaction, but that does not happen: instead, addition of increasing amounts of CNP at the start of the reaction progressively inhibits it, on the same basis as the other anions which displace hydroxide from around the cage surface. So autocatalysis is not happening here, presumably because of the poor basicity of the CNP anion.^33^ Instead we ascribe the small sigmoidal contribution in the reaction profiles when the reaction is particularly strongly inhibited to the change in *λ*_max_ of the CNP absorption maximum that we discussed earlier. A change in balance between cage-bound and free CNP as the reaction proceeds could lead to the extinction coefficient at the monitoring wavelength increasing slightly as the reaction proceeds which would give this effect. This vindicates our decision to use initial rates as the basis for comparison between the effects of different anions, which are the values used in Table 1: general trends associated with the effects of different added anions are quite clear.

## Conclusion

In contrast to our previously reported cavity-catalysed Kemp elimination using the cage **Hw** as catalyst and benzisoxazole as substrate,^6,7^ use of NBI as substrate resulted in much lower catalytic rate enhancement with the reaction occurring outside the cage cavity, at the exterior surface which is nonetheless both hydrophobic and cationic and therefore provides a locus for co-location of the hydrophobic NBI substrate and the anionic reaction partner (normally hydroxide). We could therefore use the reaction rate as a way to monitor the affinity of the cage surface for different types of added anion, as accumulation of other anions around the cage surface displaces hydroxide and slows down the surface-catalysed reactions. We observed that for a range of simple mono-anions the affinity for the cage surface followed the Hofmeister series, with more weakly solvated anions (*e.g.* bromide, nitrate) having a higher affinity for binding to the cage surface^10^ and therefore causing a greater degree of inhibition of the Kemp elimination. In opposition to this effect, if the added anions are themselves basic, their accumulation around the cage surface can accelerate the reaction to an extent related to their basicity. Although these added anions (fluoride, acetate, hydrogen carbonate *etc.*) are weaker bases than hydroxide they are present in the reaction medium at much higher concentrations than hydroxide at pH 7, resulting in a high local concentration close to the NBI substrate around the cage surface, accounting for this observation. The CNP anion itself can bind to the cage surface, more strongly than chloride but less strongly than bromide, as demonstrated by UV/Vis spectroscopy; the absence of autocatalysis suggests that it is too weakly basic to complete significantly with hydroxide.

Overall the role of the cage in bringing hydrophobic organic species and anions into proximity, which is the basis of the catalytic effects that we have previously observed,^6–9^ is very clear, which is useful to know considering the possibilities for catalysis that arise from accumulation of phenolate anions at the cage surface. The nucleophilicity of phenolate anions, for example, may provide the basis for reactions with a cavity-bound electrophile if multiple phenolates can surround a cavity-bound guest giving a high local concentration.^7^ More generally we can imagine that any of a vast range of reactions between a neutral/hydrophobic organic substrate in the cavity, and an anionic nucleophile with a high local concentration due to accumulation at the cage surface, are ripe targets to investigate for catalysis in this way.

## Experimental

Samples of **H** (used for the crystalline sponge experiment)^34^ and **Hw** (used for solution catalysis studies)^16*b*^ were prepared as previously described. Inorganic salts used to evaluate anion binding affinities were purchased from Sigma-Aldrich and used as delivered. Catalysis studies were carried out at 298 K using a BMG ClarioStar plate reader with 96-well plates by measuring UV/Vis spectra of the emerging 2-cyano-4-nitrophenolate anion (350–500 nm) and then monitoring the reaction by taking the absorbance at the maximum which shifts slightly between experiments according to the nature of other anions present (see main text). The extinction coefficient of the CNP absorption maximum was taken as 15 800 M^−1^ cm^−1^ irrespective of whether CNP is free or cage-bound; the small error introduced by this (see Fig. 6b) is subsumed in the ±5% estimated uncertainty for the rate constant values in Table 1. Samples for catalysis studies were prepared as described in the relevant figure captions and buffered at pH 7 using 16.7 mM phosphate buffer. Each dataset reported is based on the average of four individual measurements with different samples. The starting material NBI was prepared according to the literature procedure.^14*c*^ The ESI contains the data used for the rate constant calculations in Table 1.

Information on the crystal properties, data collection and refinement parameters associated with the structure determination of the **H**/**NBI** host–guest complex is collected in Table 2. The data collection was performed in Experiment Hutch 1 of beamline I-19 at the UK Diamond Light Source synchrotron facility,^35^ using methodology, data processing and software outlined previously.^19^ CCDC deposition number: 2107397.†

**Table 2:** Crystal parameters, data collection and refinement details for the structure of the **H**/**NBI** complex

Complex	**H**·**NBI1.14**
Formula	C_374.84_H_392.48_B_14.2_Cl_1.8_Co_8_F_56.8_N_74.24_O_34.36_
Molecular weight	8255.24
*T*/K	100(1)
Radiation wavelength/Å	Synchrotron (0.6889)
Crystal system	Monoclinic
Space group	*C*2/*c*
*a*/Å	33.12481(19)
*b*/Å	30.0412(2)
*c*/Å	40.1650(3)
*β*/°	95.9731(6)
*V*/Å^3^	39 751.6(3)
*Z*	4
*ρ*/g cm^−3^	1.379
Crystal size/mm^3^	0.13 × 0.10 × 0.08
*μ*/mm^−1^	0.409
Data, restraints, parameters	63 327, 6797, 2523
Final *R*_1_, w*R*_2_^a^	0.0886, 0.3254
Largest diff. peak/hole/e Å^−3^	1.33/−0.74

a The value of *R*_1_ is based on ‘observed’ data with *I* > 2*σ*(*I*); the value of w*R*_2_ is based on all data.

## Data availability

Data that is not in the ESI is available from the corresponding author (MDW) on request.

## Author contributions

Synthesis: MDL, JST. Binding constant measurement: JST. X-Ray crystallography: CGPT. Plate reader and UV/Vis spectroscopic measurements: MDL, MBT, JD, KLT. Data analysis: MDL, NHW, KLT. Manuscript preparation: MDW, NHW, MDL. Project conception and supervision: MDW.

## Conflicts of interest

There are no conflicts to declare.

## Supplementary Material

SC-012-D1SC04887B-s001

SC-012-D1SC04887B-s002
