# Supplementary material for: Interaction of anions with the surface of a coordination cage in aqueous solution probed by their effect on a cage-catalysed Kemp elimination
Source: Chem Sci. 2021 Oct 25;12(44):14781–91. doi: 10.1039/d1sc04887b (PMC8597839; doi:10.1039/d1sc04887b)
Supplement: SC-012-D1SC04887B-s001 [file SC-012-D1SC04887B-s001.pdf]

Electronic Supplementary Material (ESI) for Chemical Science.
This journal is © The Royal Society of Chemistry 2021

Interaction of anions with the surface of a coordination cage in aqueous solution probed by their effect on a cage-catalysed Kemp elimination

Michael D. Ludden, Christopher G. P. Taylor, Max B. Tipping, Jennifer S. Train, Nicholas H. Williams, Jack C. Dorrat, Kellie L. Tuck and Michael D. Ward

Supporting Information

1. Characterisation of the cage $\mathbf{H}^{\mathbf{W}}$
2. UV/vis data for control experiments
3. UV/vis data for anion addition experiments
4. UV/vis data for anions of varying basicity experiments
5. Additional figures

1. Characterisation of the cage $\mathbf{H^W}$

The cage $\mathbf{H^W}$ was prepared following a previously described procedure.[S1] After dissolution in deionised water, samples of $\mathbf{H^W}$ were filtered using a 0.2 µm Millipore filter to remove any solid particulates. The pH of the cage solution was then adjusted to pH 7 through addition of 1 M NaOH solution.

To confirm the stability of the cage in aqueous solution, a combination of paramagnetic ^{1}H NMR spectroscopy and electrospray mass spectrometry were used. The spectrum of $\mathbf{H^W}$ (0.2 mM, D_2O) is shown below (Figure S1).[S1] This corresponds to known spectra of the cage and confirms its formation in solution. ESI-MS results also corroborated these findings, with signals for $\mathbf{H^W}$ corresponding to loss of 7 or 8 tetrafluoroborate anions, $[\mathbf{H^W} - 7BF_4]^{7+}$ and $[\mathbf{H^W} - 8BF_4]^{8+}$ state being apparent.

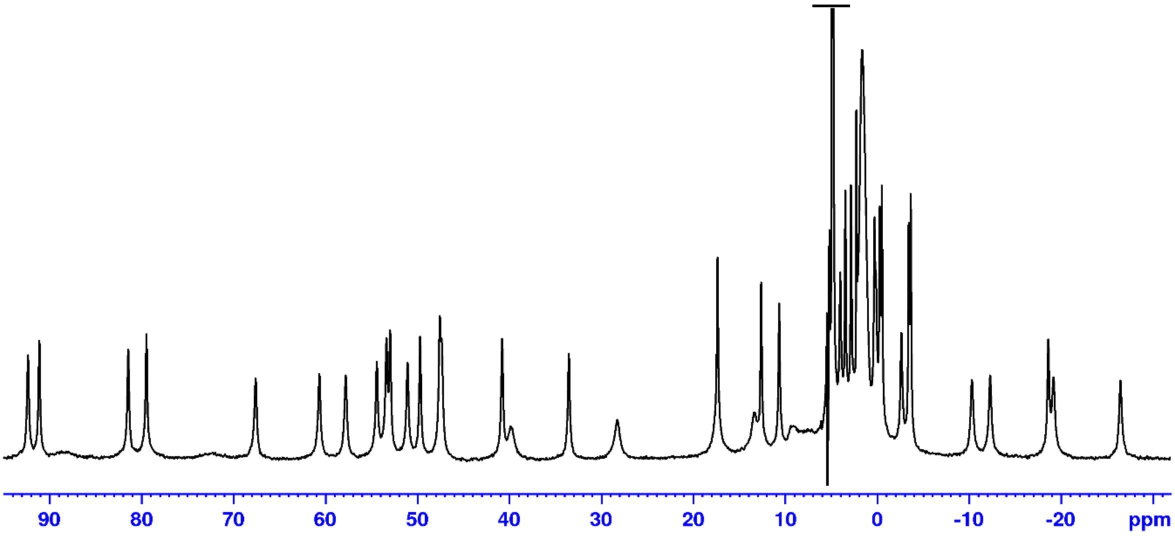

Figure S1 – ^{1}H NMR spectrum of the cage $\mathbf{H^W}$ recorded in D_2O after dissolution and subsequent filtration. T = 298 K.

2. UV/vis spectroscopic data for control experiments

2.1. Data Manipulation

From the UV/vis spectroscopy plate-readter data, values for k_{cat} and k_2 were calculated using initial rates. To do this, the absorbance at a chosen wavelength was monitored over time, with the data being recorded typically every 120 seconds for 4h, with the first 20 points being used. The wavelength chosen depends on whether the product, 2-cyano-4-nitrophenolate, is bound to the exterior of $\mathbf{H^W}$, as this induces a red-shift in the absorption spectrum (see main text). Two examples of spectra are given below, one with $\mathbf{H^W}$ present and one without.

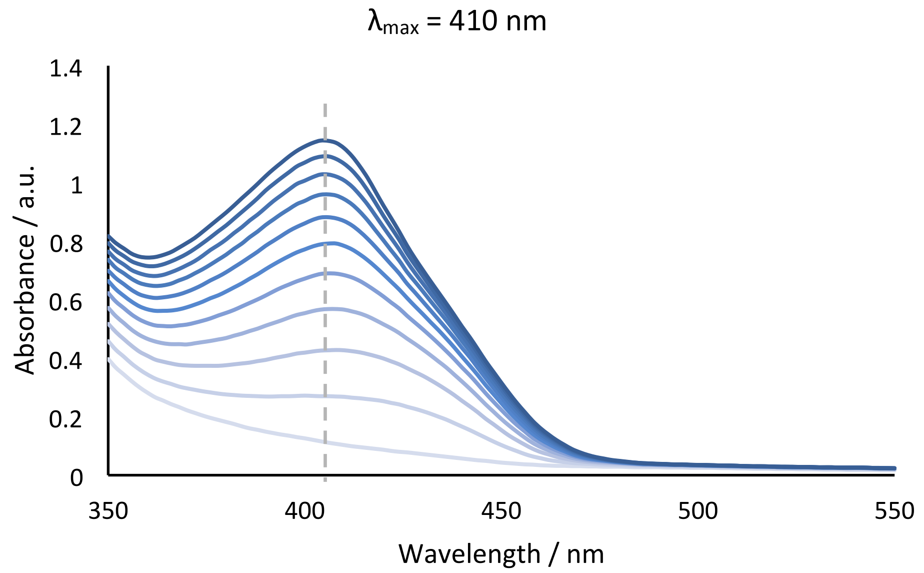

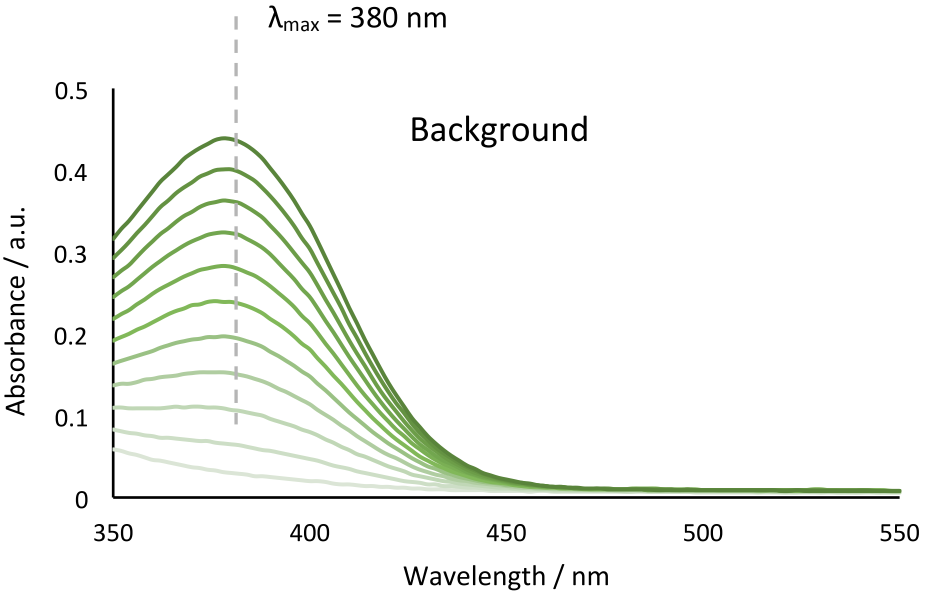

For any reactions monitored over time by UV/vis, the rate was determined through monitoring the absorbance of the product 2-cyano-4-nitrophenolate at its absorption maximum, which varies depending on the concentration of $\mathbf{H^W}$. The extinction coefficient quoted in the literature is 15,800 M^{-1} cm^{-1} (reference S2) and using this a concentration of product could be calculated from the absorbance. Each dataset was corrected by first setting the absorbance at t = 0 s to equal zero, followed by subtracting the background absorbance from the uncatalysed reaction under the same conditions at each data point to give the catalysed reaction profile. Taking the natural log of the concentration of starting material and using the LINEST function in Excel, a rate constant could be determined for each dataset along with the standard error for the data. This analysis function was typically used over the first 20 datapoints, giving an initial rate. Each set of values for absorbance was an average of 4 repeats.

A summary of the experiments covered in Section 2 of this Supporting Information is provided below. As described above, values for k_{cat} and the SE of k_{cat} were obtained using the LINEST function and are provided for each dataset.

Sample	Sample name	λ_{max}	$[H^W]$ / µM	k_{cat} / s^{-1}	SE of k_{cat}	% error	k_2 / M^{-1} s^{-1}
X1	Background	380	0	1.47E-05	2.34E-07	1.6	N/A
X2	H^W @ 67 µM	404	66.7	7.84E-05	6.28E-07	0.8	1.18
X3	H^W @ 33 µM	396	33.3	4.32E-05	5.77E-07	1.3	1.30
X4	H^W @ 16.7 µM	388	16.67	2.19E-05	9.89E-08	0.5	1.31
X5	H^W @ 6.7 µM	382	6.67	9.53E-06	1.2E-07	1.3	1.43

The data presented on the following pages is both tabulated and graphical representations of the calculations for initial rates.

2.2. Catalysis of NBI to 2CNP under **background** conditions (0.25 mM NBI, 16.7 mM pH 7 phosphate buffer, no **H**^{W}). λ_{max} = 380 nm.

Time	Abs	corrected abs	[2CP] / M	[NBI] / M	ln NBI
0	0.076	0	0.00E+00	2.00E-04	-8.517
120	0.08	0.004	2.53E-07	2.00E-04	-8.518
240	0.084	0.008	5.06E-07	1.99E-04	-8.520
360	0.088	0.012	7.59E-07	1.99E-04	-8.521
480	0.093	0.017	1.08E-06	1.99E-04	-8.523
600	0.099	0.023	1.46E-06	1.99E-04	-8.524
720	0.103	0.027	1.71E-06	1.98E-04	-8.526
840	0.108	0.032	2.03E-06	1.98E-04	-8.527
960	0.113	0.037	2.34E-06	1.98E-04	-8.529
1080	0.119	0.043	2.72E-06	1.97E-04	-8.531
1200	0.124	0.048	3.04E-06	1.97E-04	-8.532
1320	0.131	0.055	3.48E-06	1.97E-04	-8.535
1440	0.136	0.06	3.80E-06	1.96E-04	-8.536
1560	0.142	0.066	4.18E-06	1.96E-04	-8.538
1680	0.148	0.072	4.56E-06	1.95E-04	-8.540
1800	0.154	0.078	4.94E-06	1.95E-04	-8.542
1920	0.161	0.085	5.38E-06	1.95E-04	-8.544
2040	0.167	0.091	5.76E-06	1.94E-04	-8.546
2160	0.173	0.097	6.14E-06	1.94E-04	-8.548
2280	0.179	0.103	6.52E-06	1.93E-04	-8.550

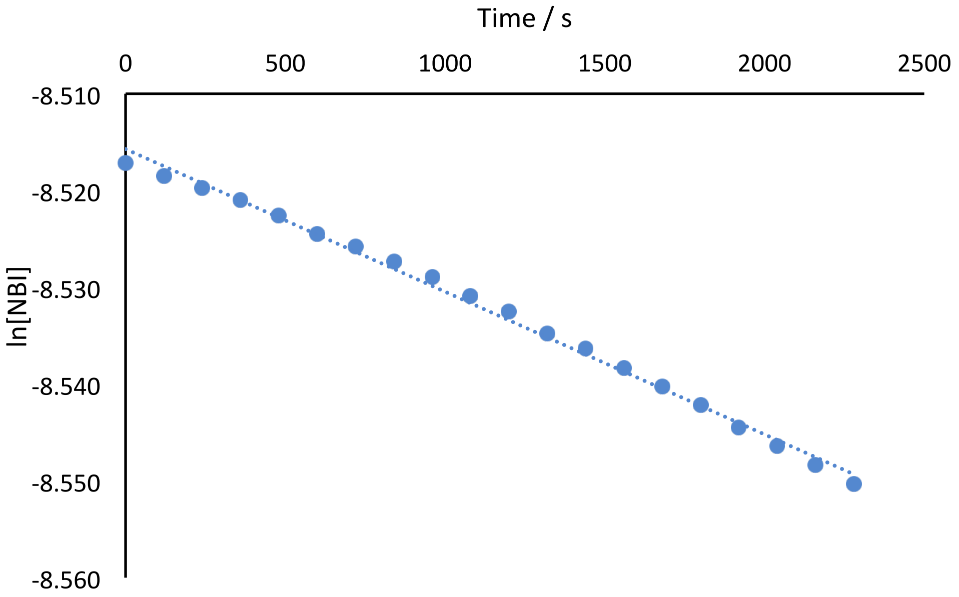

slope	-1.47E-05	-8.52	intercept
SE slope	2.34E-07	3.12E-04	SE intercept
R2	0.995	7.23E-04	SE (y)
F	3975	18	df
SS reg	2.08E-03	9.42E-06	SSR or SSE

2.3. Catalysis of NBI to 2CNP with **67 µM** cage added (0.25 mM NBI, 16.7 mM pH 7 phosphate buffer, 67 µM $\mathbf{H}^{W}$). λ_{max} = 404 nm.

Time	Abs	correct to 0	minus bk	[2CNP] / M	[NBI] / M	ln NBI
0	0.211	0	0	0.00E+00	2.00E-04	-8.517
120	0.247	0.036	0.032	2.03E-06	1.98E-04	-8.527
240	0.282	0.071	0.063	3.99E-06	1.96E-04	-8.537
360	0.317	0.106	0.094	5.95E-06	1.94E-04	-8.547
480	0.351	0.14	0.123	7.78E-06	1.92E-04	-8.557
600	0.387	0.176	0.153	9.68E-06	1.90E-04	-8.567
720	0.422	0.211	0.184	1.16E-05	1.88E-04	-8.577
840	0.457	0.246	0.214	1.35E-05	1.86E-04	-8.587
960	0.49	0.279	0.242	1.53E-05	1.85E-04	-8.597
1080	0.524	0.313	0.27	1.71E-05	1.83E-04	-8.607
1200	0.557	0.346	0.298	1.89E-05	1.81E-04	-8.616
1320	0.59	0.379	0.324	2.05E-05	1.79E-04	-8.625
1440	0.622	0.411	0.351	2.22E-05	1.78E-04	-8.635
1560	0.653	0.442	0.376	2.38E-05	1.76E-04	-8.644
1680	0.683	0.472	0.4	2.53E-05	1.75E-04	-8.653
1800	0.715	0.504	0.426	2.70E-05	1.73E-04	-8.662
1920	0.744	0.533	0.448	2.84E-05	1.72E-04	-8.670
2040	0.772	0.561	0.47	2.97E-05	1.70E-04	-8.678
2160	0.8	0.589	0.492	3.11E-05	1.69E-04	-8.686
2280	0.828	0.617	0.514	3.25E-05	1.67E-04	-8.695

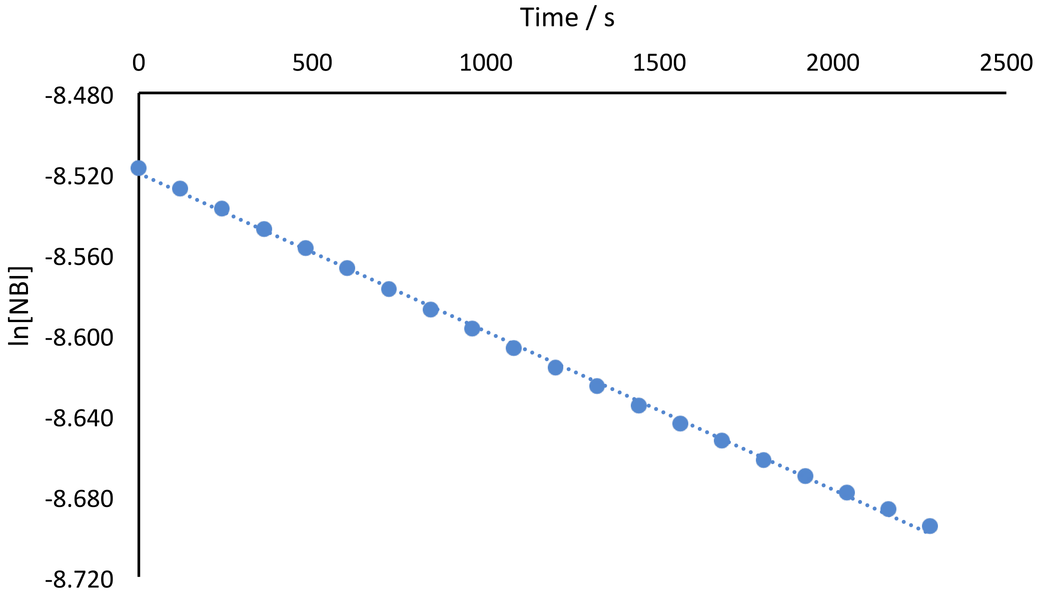

slope	-7.84E-05	-8.52	intercept
SE slope	6.28E-07	8.38E-04	SE intercept
R2	0.999	1.94E-03	SE (y)
F	15563	18	df
SS reg	5.89E-02	6.81E-05	SSR or SSE

2.4. Catalysis of NBI to 2CNP with **33 µM** cage added (0.25 mM NBI, 16.7 mM pH 7 phosphate buffer, 33 µM $\mathbf{H}^{W}$). λ_{max} = 396 nm.

Time	Abs	correct to 0	minus bk	[2CNP] / M	[NBI] / M	ln NBI
0	0.132	0	0	0.00E+00	2.00E-04	-8.517
120	0.155	0.023	0.019	1.20E-06	1.99E-04	-8.523
240	0.175	0.043	0.035	2.22E-06	1.98E-04	-8.528
360	0.202	0.07	0.058	3.67E-06	1.96E-04	-8.536
480	0.228	0.096	0.079	5.00E-06	1.95E-04	-8.543
600	0.25	0.118	0.095	6.01E-06	1.94E-04	-8.548
720	0.271	0.139	0.112	7.09E-06	1.93E-04	-8.553
840	0.293	0.161	0.129	8.16E-06	1.92E-04	-8.559
960	0.315	0.183	0.146	9.24E-06	1.91E-04	-8.564
1080	0.336	0.204	0.161	1.02E-05	1.90E-04	-8.569
1200	0.354	0.222	0.174	1.10E-05	1.89E-04	-8.574
1320	0.373	0.241	0.186	1.18E-05	1.88E-04	-8.578
1440	0.393	0.261	0.201	1.27E-05	1.87E-04	-8.583
1560	0.414	0.282	0.216	1.37E-05	1.86E-04	-8.588
1680	0.435	0.303	0.231	1.46E-05	1.85E-04	-8.593
1800	0.455	0.323	0.245	1.55E-05	1.84E-04	-8.598
1920	0.475	0.343	0.258	1.63E-05	1.84E-04	-8.602
2040	0.495	0.363	0.272	1.72E-05	1.83E-04	-8.607
2160	0.515	0.383	0.286	1.81E-05	1.82E-04	-8.612
2280	0.536	0.404	0.301	1.91E-05	1.81E-04	-8.617

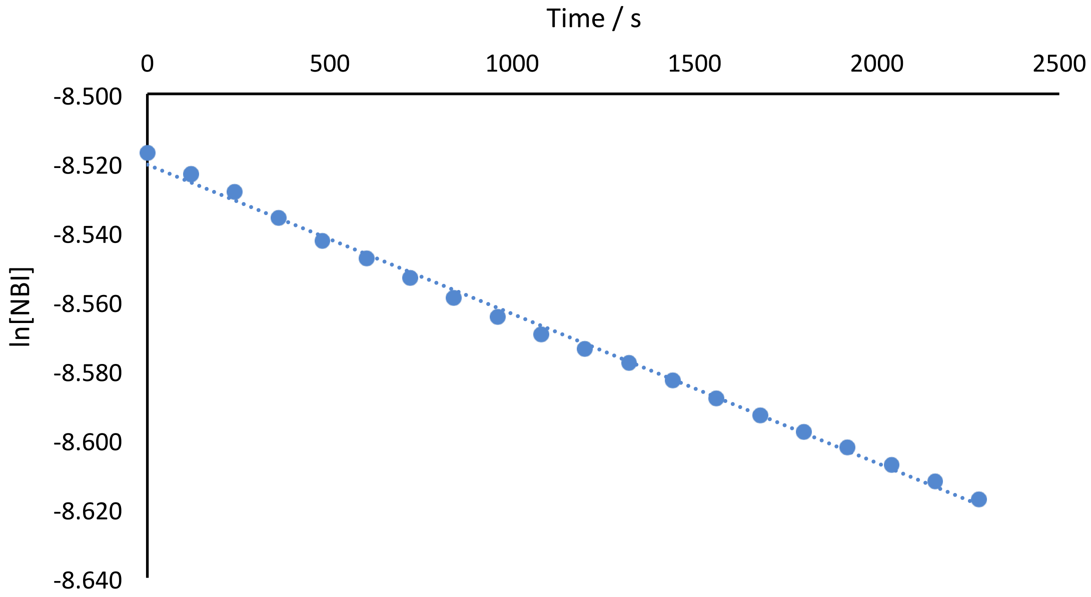

slope	-4.32E-05	-8.52	intercept
SE slope	5.77E-07	7.69E-04	SE intercept
R2	0.997	1.78E-03	SE (y)
F	5598	18	df
SS reg	1.78E-02	5.73E-05	SSR or SSE

2.5. Catalysis of NBI to 2CNP with **16.7 µM** cage added (0.25 mM NBI, 16.7 mM pH 7 phosphate buffer, 16.7 µM $\mathbf{H^{W}}$). λ_{max} = 388 nm.

Time	Abs	correct to 0	minus bk	[2CNP] / M	[NBI] / M	ln NBI
0	0.146	0	0	0.00E+00	2.00E-04	-8.517
120	0.157	0.011	0.007	4.43E-07	2.00E-04	-8.519
240	0.169	0.023	0.015	9.49E-07	1.99E-04	-8.522
360	0.182	0.036	0.024	1.52E-06	1.98E-04	-8.525
480	0.194	0.048	0.031	1.96E-06	1.98E-04	-8.527
600	0.209	0.063	0.04	2.53E-06	1.97E-04	-8.530
720	0.221	0.075	0.048	3.04E-06	1.97E-04	-8.532
840	0.235	0.089	0.057	3.61E-06	1.96E-04	-8.535
960	0.249	0.103	0.066	4.18E-06	1.96E-04	-8.538
1080	0.263	0.117	0.074	4.68E-06	1.95E-04	-8.541
1200	0.276	0.13	0.082	5.19E-06	1.95E-04	-8.543
1320	0.291	0.145	0.09	5.70E-06	1.94E-04	-8.546
1440	0.305	0.159	0.099	6.27E-06	1.94E-04	-8.549
1560	0.319	0.173	0.107	6.77E-06	1.93E-04	-8.552
1680	0.332	0.186	0.114	7.22E-06	1.93E-04	-8.554
1800	0.346	0.2	0.122	7.72E-06	1.92E-04	-8.557
1920	0.36	0.214	0.129	8.16E-06	1.92E-04	-8.559
2040	0.374	0.228	0.137	8.67E-06	1.91E-04	-8.562
2160	0.387	0.241	0.144	9.11E-06	1.91E-04	-8.564
2280	0.401	0.255	0.152	9.62E-06	1.90E-04	-8.566

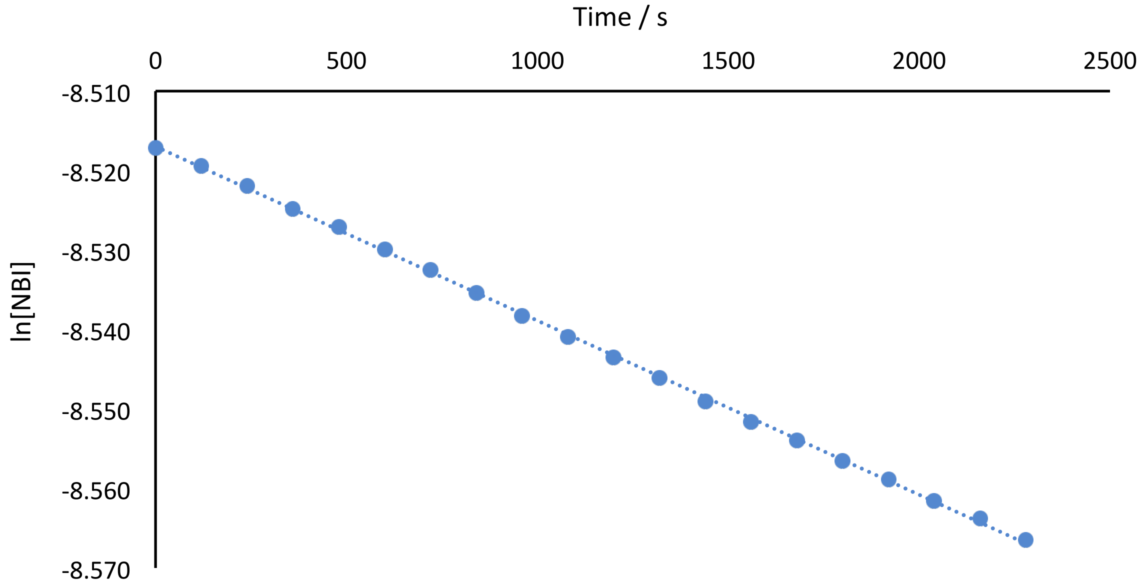

slope	-2.19E-05	-8.52	intercept
SE slope	9.89E-08	1.32E-04	SE intercept
R2	1.000	3.06E-04	SE (y)
F	49079	18	df
SS reg	4.60E-03	1.69E-06	SSR or SSE

2.6. Catalysis of NBI to 2CNP with **6.7 µM cage** added (0.25 mM NBI, 16.7 mM pH 7 phosphate buffer, 6.7 µM $\mathbf{H}^{W}$). λ_{max} = 382 nm.

Time	Abs	correct to 0	minus bk	[2CNP] / M	[NBI] / M	ln NBI
0	0.045	0	0	0.00E+00	2.00E-04	-8.517
120	0.052	0.007	0.003	1.90E-07	2.00E-04	-8.518
240	0.059	0.014	0.006	3.80E-07	2.00E-04	-8.519
360	0.066	0.021	0.009	5.70E-07	1.99E-04	-8.520
480	0.074	0.029	0.012	7.59E-07	1.99E-04	-8.521
600	0.082	0.037	0.014	8.86E-07	1.99E-04	-8.522
720	0.09	0.045	0.018	1.14E-06	1.99E-04	-8.523
840	0.099	0.054	0.022	1.39E-06	1.99E-04	-8.524
960	0.108	0.063	0.026	1.65E-06	1.98E-04	-8.525
1080	0.118	0.073	0.03	1.90E-06	1.98E-04	-8.527
1200	0.126	0.081	0.033	2.09E-06	1.98E-04	-8.528
1320	0.136	0.091	0.036	2.28E-06	1.98E-04	-8.529
1440	0.145	0.1	0.04	2.53E-06	1.97E-04	-8.530
1560	0.155	0.11	0.044	2.78E-06	1.97E-04	-8.531
1680	0.165	0.12	0.048	3.04E-06	1.97E-04	-8.532
1800	0.175	0.13	0.052	3.29E-06	1.97E-04	-8.534
1920	0.185	0.14	0.055	3.48E-06	1.97E-04	-8.535
2040	0.195	0.15	0.059	3.73E-06	1.96E-04	-8.536
2160	0.205	0.16	0.063	3.99E-06	1.96E-04	-8.537
2280	0.216	0.171	0.068	4.30E-06	1.96E-04	-8.539

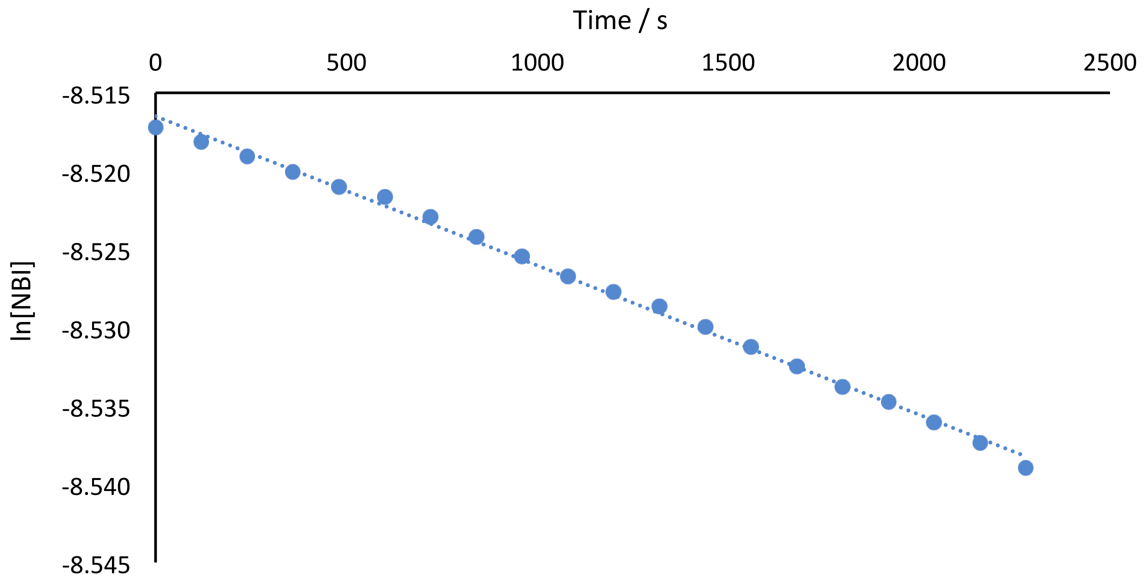

slope	-9.53E-06	-8.52	intercept
SE slope	1.20E-07	1.61E-04	SE intercept
R2	0.997	3.72E-04	SE (y)
F	6267	18	df
SS reg	8.70E-04	2.50E-06	SSR or SSE

3. UV/vis spectroscopic data for anion addition experiments

3.1. Summary of experiments

All data was obtained as described in Section 3.1.

**Anion**	**λ_{max} / nm**	**k_{cat} / s-1**	**SE in k_{cat}**	**% error**	**k_2 / M^{-1} s^{-1}**
Background	380	1.80E-05	2.76E-07	1.5	
H^W only	410	1.35E-04	9.97E-07	0.7	1.08
F^-	410	1.42E-04	1.08E-06	0.8	1.14
Cl^-	410	1.01E-04	2.80E-07	0.3	0.81
Br^-	392	3.58E-05	6.37E-07	1.8	0.29
NO_3^-	380	2.92E-05	4.96E-07	1.7	0.23
SO_4^{2-}	410	1.07E-04	5.52E-07	0.5	0.85
$IO3^-$	410	1.12E-04	1.10E-06	1.0	0.89
HCO_3^-	410	2.33E-04	5.46E-06	2.3	1.86

3.2. Conversion of NBI to 2CNP under **background** conditions (0.2 mM NBI, 16.7 mM pH 7 phosphate buffer, no **H**^{W}). λ_{max} = 380 nm.

**Time / s**	**Abs / a.u.**	**zero'd**	**[2CNP] / M**	**[NBI] / M**	**ln(NBI)**
0	0.045	0	0.00E+00	2.00E-04	-8.517
120	0.051	0.006	3.80E-07	2.00E-04	-8.519
240	0.055	0.01	6.33E-07	1.99E-04	-8.520
360	0.061	0.016	1.01E-06	1.99E-04	-8.522
480	0.068	0.023	1.46E-06	1.99E-04	-8.524
600	0.072	0.027	1.71E-06	1.98E-04	-8.526
720	0.079	0.034	2.15E-06	1.98E-04	-8.528
840	0.085	0.04	2.53E-06	1.97E-04	-8.530
960	0.092	0.047	2.97E-06	1.97E-04	-8.532
1080	0.098	0.053	3.35E-06	1.97E-04	-8.534
1200	0.107	0.062	3.92E-06	1.96E-04	-8.537
1320	0.113	0.068	4.30E-06	1.96E-04	-8.539
1440	0.12	0.075	4.75E-06	1.95E-04	-8.541
1560	0.126	0.081	5.13E-06	1.95E-04	-8.543
1680	0.134	0.089	5.63E-06	1.94E-04	-8.546
1800	0.142	0.097	6.14E-06	1.94E-04	-8.548
1920	0.15	0.105	6.65E-06	1.93E-04	-8.551
2040	0.157	0.112	7.09E-06	1.93E-04	-8.553
2160	0.164	0.119	7.53E-06	1.92E-04	-8.556

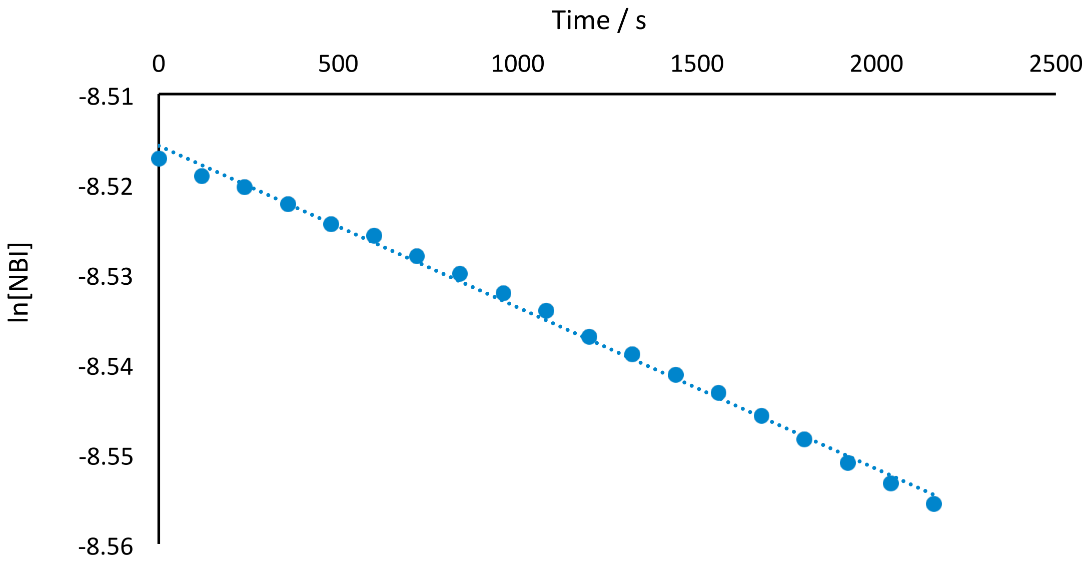

slope	-1.80E-05	-8.52	intercept
SE slope	2.76E-07	3.48E-04	SE intercept
R2	0.996	7.90E-04	SE (y)
F	4246	17	df
SS reg	2.65E-03	1.06E-05	SSR or SSE

3.3. Catalysis of NBI to 2CNP with cage added but **no additional anions** (0.2 mM NBI, 16.7 mM pH 7 phosphate buffer, 0.125 mM $\mathbf{H}^{W}$). λ_{max} = 410 nm.

Time / s	Abs / a.u.	zero'd	minus bk	[2CNP] / M	[NBI] / M	ln(NBI)
0	0.26	0	0	0.00E+00	2.00E-04	-8.517
120	0.315	0.055	0.049	3.10E-06	1.97E-04	-8.533
240	0.372	0.112	0.102	6.46E-06	1.94E-04	-8.550
360	0.428	0.168	0.152	9.62E-06	1.90E-04	-8.566
480	0.485	0.225	0.202	1.28E-05	1.87E-04	-8.583
600	0.54	0.28	0.253	1.60E-05	1.84E-04	-8.601
720	0.596	0.336	0.302	1.91E-05	1.81E-04	-8.618
840	0.65	0.39	0.35	2.22E-05	1.78E-04	-8.635
960	0.705	0.445	0.398	2.52E-05	1.75E-04	-8.652
1080	0.757	0.497	0.444	2.81E-05	1.72E-04	-8.669
1200	0.807	0.547	0.485	3.07E-05	1.69E-04	-8.684
1320	0.858	0.598	0.53	3.35E-05	1.66E-04	-8.701
1440	0.907	0.647	0.572	3.62E-05	1.64E-04	-8.717
1560	0.954	0.694	0.613	3.88E-05	1.61E-04	-8.733
1680	0.999	0.739	0.65	4.11E-05	1.59E-04	-8.747
1800	1.044	0.784	0.687	4.35E-05	1.57E-04	-8.762
1920	1.087	0.827	0.722	4.57E-05	1.54E-04	-8.777
2040	1.129	0.869	0.757	4.79E-05	1.52E-04	-8.791
2160	1.168	0.908	0.789	4.99E-05	1.50E-04	-8.804

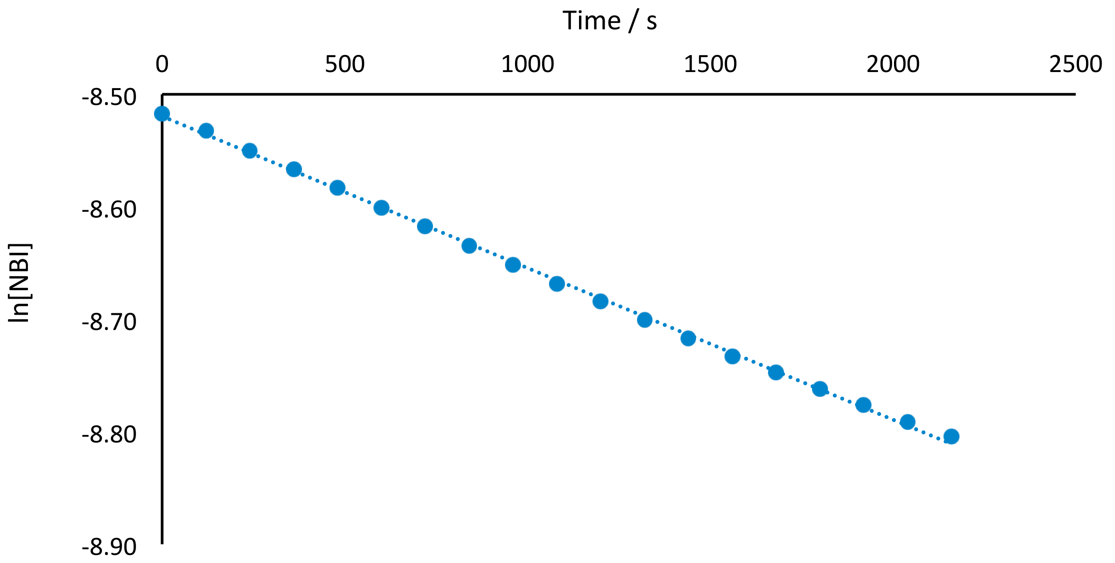

slope	-1.35E-04	-8.52	intercept
SE slope	9.97E-07	1.26E-03	SE intercept
R2	0.999	2.86E-03	SE (y)
F	18279	17	df
SS reg	1.49E-01	1.39E-04	SSR or SSE

3.4. Catalysis of NBI to 2CNP with cage and **1.67 mM fluoride** added (0.2 mM NBI, 16.7 mM pH 7 phosphate buffer, 0.125 mM $\mathbf{H^W}$). λ_{max} = 410 nm.

Time / s	Abs / a.u.	zero'd	minus bk	[2CNP] / M	[NBI] / M	ln(NBI)
0	0.274	0	0	0.00E+00	2.00E-04	-8.517
120	0.334	0.06	0.054	3.42E-06	1.97E-04	-8.534
240	0.395	0.121	0.111	7.03E-06	1.93E-04	-8.553
360	0.455	0.181	0.165	1.04E-05	1.90E-04	-8.571
480	0.513	0.239	0.216	1.37E-05	1.86E-04	-8.588
600	0.572	0.298	0.271	1.72E-05	1.83E-04	-8.607
720	0.63	0.356	0.322	2.04E-05	1.80E-04	-8.625
840	0.686	0.412	0.372	2.35E-05	1.76E-04	-8.642
960	0.742	0.468	0.421	2.66E-05	1.73E-04	-8.660
1080	0.797	0.523	0.47	2.97E-05	1.70E-04	-8.678
1200	0.848	0.574	0.512	3.24E-05	1.68E-04	-8.694
1320	0.901	0.627	0.559	3.54E-05	1.65E-04	-8.712
1440	0.952	0.678	0.603	3.82E-05	1.62E-04	-8.729
1560	1.001	0.727	0.646	4.09E-05	1.59E-04	-8.746
1680	1.048	0.774	0.685	4.34E-05	1.57E-04	-8.762
1800	1.095	0.821	0.724	4.58E-05	1.54E-04	-8.777
1920	1.14	0.866	0.761	4.82E-05	1.52E-04	-8.793
2040	1.181	0.907	0.795	5.03E-05	1.50E-04	-8.807
2160	1.222	0.948	0.829	5.25E-05	1.48E-04	-8.821

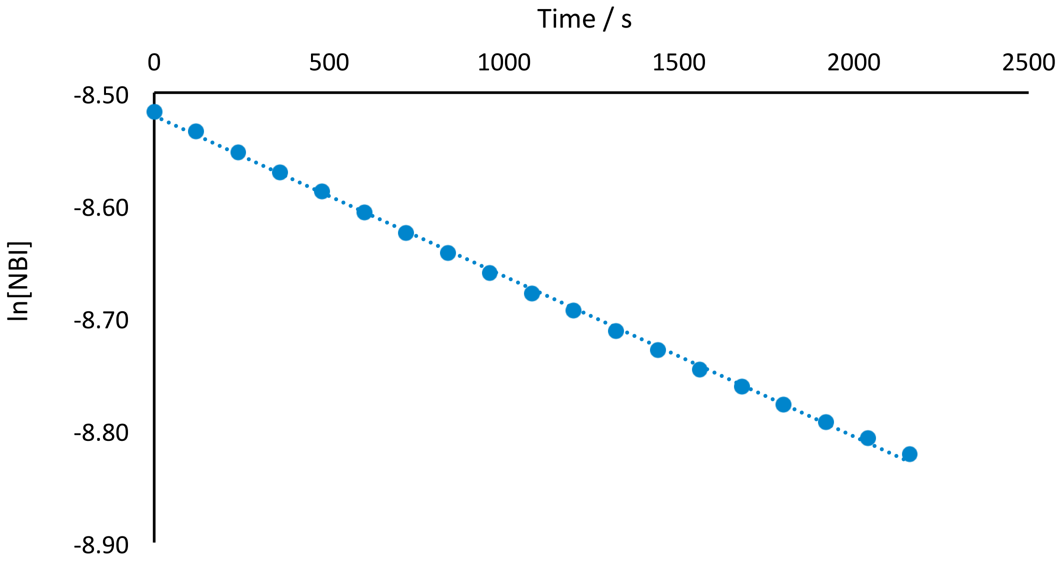

slope	-1.42E-04	-8.52	intercept
SE slope	1.08E-06	1.36E-03	SE intercept
R2	0.999	3.09E-03	SE (y)
F	17451	17	df
SS reg	1.67E-01	1.62E-04	SSR or SSE

3.5. Catalysis of NBI to 2CNP with cage and **1.67 mM chloride** added (0.2 mM NBI, 16.7 mM pH 7 phosphate buffer, 0.125 mM $\mathbf{H}^{W}$). λ_{max} = 410 nm.

Time / s	Abs / a.u.	zero'd	minus bk	[2CNP] / M	[NBI] / M	ln(NBI)
0	0.226	0	0	0.00E+00	2.00E-04	-8.517
120	0.267	0.041	0.035	2.22E-06	1.98E-04	-8.528
240	0.307	0.081	0.071	4.49E-06	1.96E-04	-8.540
360	0.349	0.123	0.107	6.77E-06	1.93E-04	-8.552
480	0.391	0.165	0.142	8.99E-06	1.91E-04	-8.563
600	0.433	0.207	0.18	1.14E-05	1.89E-04	-8.576
720	0.476	0.25	0.216	1.37E-05	1.86E-04	-8.588
840	0.518	0.292	0.252	1.59E-05	1.84E-04	-8.600
960	0.561	0.335	0.288	1.82E-05	1.82E-04	-8.613
1080	0.604	0.378	0.325	2.06E-05	1.79E-04	-8.626
1200	0.645	0.419	0.357	2.26E-05	1.77E-04	-8.637
1320	0.686	0.46	0.392	2.48E-05	1.75E-04	-8.650
1440	0.727	0.501	0.426	2.70E-05	1.73E-04	-8.662
1560	0.77	0.544	0.463	2.93E-05	1.71E-04	-8.676
1680	0.806	0.58	0.491	3.11E-05	1.69E-04	-8.686
1800	0.845	0.619	0.522	3.30E-05	1.67E-04	-8.698
1920	0.883	0.657	0.552	3.49E-05	1.65E-04	-8.709
2040	0.925	0.699	0.587	3.72E-05	1.63E-04	-8.723
2160	0.961	0.735	0.616	3.90E-05	1.61E-04	-8.734

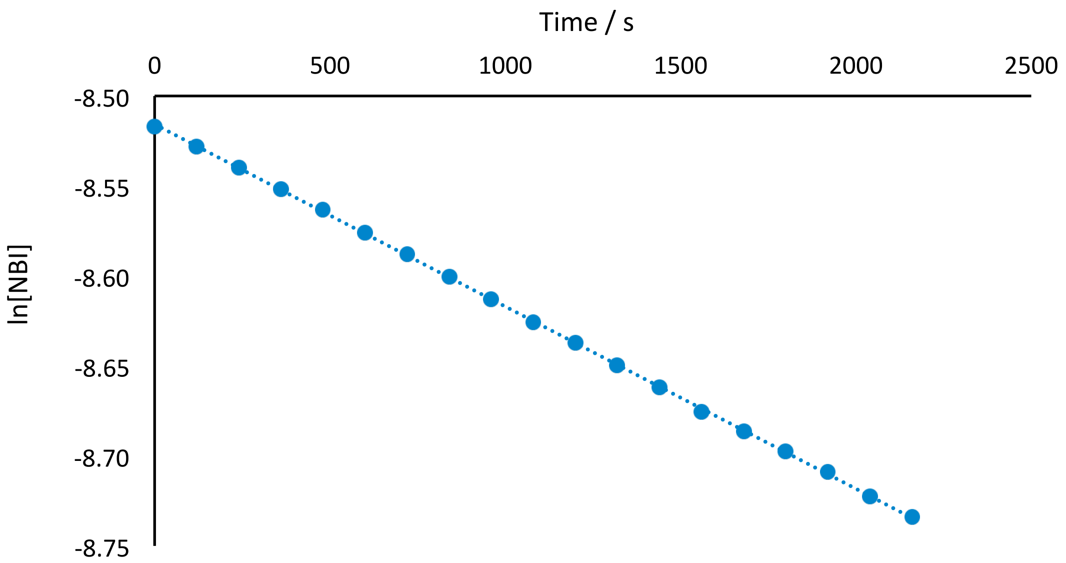

slope	-1.01E-04	-8.52	intercept
SE slope	2.80E-07	3.53E-04	SE intercept
R2	1.000	8.01E-04	SE (y)
F	131248	17	df
SS reg	8.42E-02	1.09E-05	SSR or SSE

3.6. Catalysis of NBI to 2CNP with cage and **1.67 mM bromide** added (0.2 mM NBI, 16.7 mM pH 7 phosphate buffer, 0.125 mM $\mathbf{H^W}$). λ_{max} = 392 nm.

Time / s	Abs / a.u.	zero'd	minus bk	[2CNP] / M	[NBI] / M	ln(NBI)
0	0.282	0	0	0.00E+00	2.00E-04	-8.517
120	0.296	0.014	0.008	5.06E-07	1.99E-04	-8.520
240	0.311	0.029	0.019	1.20E-06	1.99E-04	-8.523
360	0.327	0.045	0.029	1.84E-06	1.98E-04	-8.526
480	0.344	0.062	0.039	2.47E-06	1.98E-04	-8.530
600	0.362	0.08	0.053	3.35E-06	1.97E-04	-8.534
720	0.379	0.097	0.063	3.99E-06	1.96E-04	-8.537
840	0.399	0.117	0.077	4.87E-06	1.95E-04	-8.542
960	0.419	0.137	0.09	5.70E-06	1.94E-04	-8.546
1080	0.438	0.156	0.103	6.52E-06	1.93E-04	-8.550
1200	0.458	0.176	0.114	7.22E-06	1.93E-04	-8.554
1320	0.479	0.197	0.129	8.16E-06	1.92E-04	-8.559
1440	0.501	0.219	0.144	9.11E-06	1.91E-04	-8.564
1560	0.524	0.242	0.161	1.02E-05	1.90E-04	-8.569
1680	0.545	0.263	0.174	1.10E-05	1.89E-04	-8.574
1800	0.567	0.285	0.188	1.19E-05	1.88E-04	-8.579
1920	0.589	0.307	0.202	1.28E-05	1.87E-04	-8.583
2040	0.611	0.329	0.217	1.37E-05	1.86E-04	-8.588
2160	0.633	0.351	0.232	1.47E-05	1.85E-04	-8.593

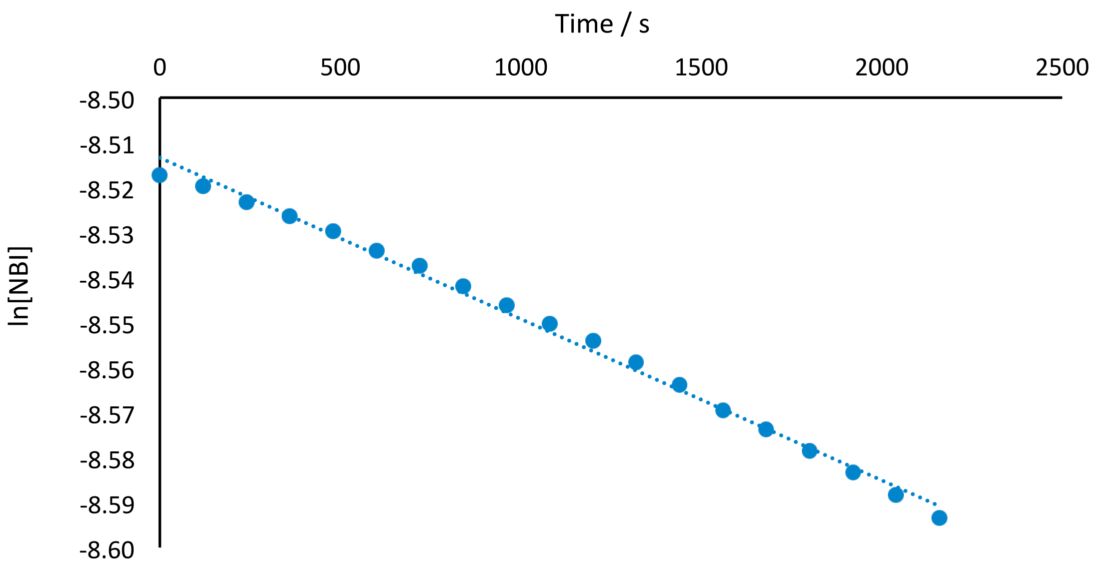

slope	-3.58E-05	-8.51	intercept
SE slope	6.37E-07	8.05E-04	SE intercept
R2	0.995	1.82E-03	SE (y)
F	3170	17	df
SS reg	1.05E-02	5.657E-05	SSR or SSE

3.7. Catalysis of NBI to 2CNP with cage and **1.67 mM nitrate** added (0.2 mM NBI, 16.7 mM pH 7 phosphate buffer, 0.125 mM $\mathbf{H^{W}}$). λ_{max} = 380 nm.

Time / s	Abs / a.u.	zero'd	minus bk	[2CNP] / M	[NBI] / M	ln(NBI)
0	0.362	0	0	0.00E+00	2.00E-04	-8.517
120	0.375	0.013	0.007	4.43E-07	2.00E-04	-8.519
240	0.388	0.026	0.016	1.01E-06	1.99E-04	-8.522
360	0.402	0.04	0.024	1.52E-06	1.98E-04	-8.525
480	0.416	0.054	0.031	1.96E-06	1.98E-04	-8.527
600	0.432	0.07	0.043	2.72E-06	1.97E-04	-8.531
720	0.45	0.088	0.054	3.42E-06	1.97E-04	-8.534
840	0.464	0.102	0.062	3.92E-06	1.96E-04	-8.537
960	0.482	0.12	0.073	4.62E-06	1.95E-04	-8.541
1080	0.502	0.14	0.087	5.51E-06	1.94E-04	-8.545
1200	0.527	0.165	0.103	6.52E-06	1.93E-04	-8.550
1320	0.548	0.186	0.118	7.47E-06	1.93E-04	-8.555
1440	0.555	0.193	0.118	7.47E-06	1.93E-04	-8.555
1560	0.576	0.214	0.133	8.42E-06	1.92E-04	-8.560
1680	0.591	0.229	0.14	8.86E-06	1.91E-04	-8.563
1800	0.612	0.25	0.153	9.68E-06	1.90E-04	-8.567
1920	0.635	0.273	0.168	1.06E-05	1.89E-04	-8.572
2040	0.65	0.288	0.176	1.11E-05	1.89E-04	-8.575
2160	0.669	0.307	0.188	1.19E-05	1.88E-04	-8.579

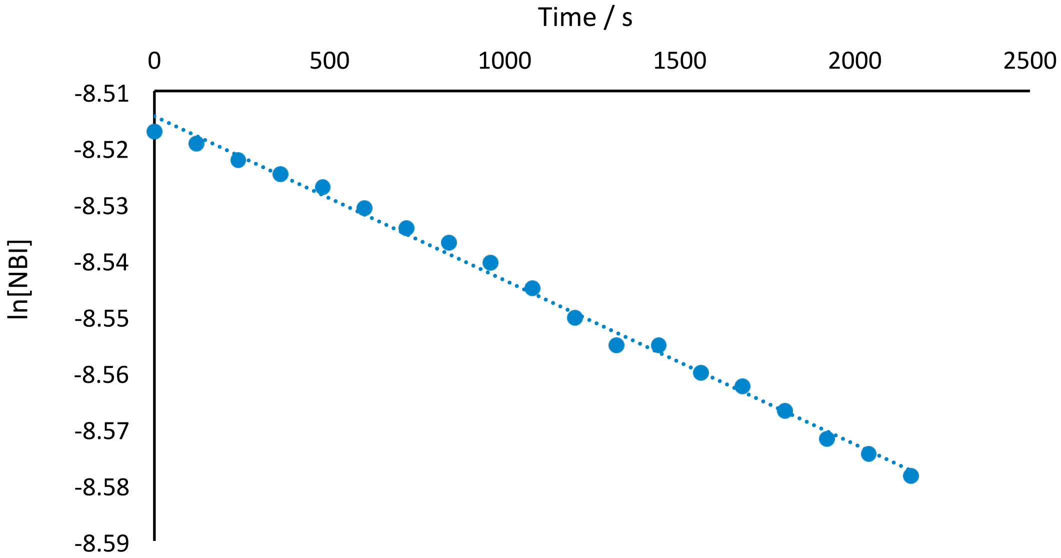

slope	-2.92E-05	-8.51	intercept
SE slope	4.96E-07	6.27E-04	SE intercept
R2	0.995	1.42E-03	SE (y)
F	3464	17	df
SS reg	6.98E-03	3.43E-05	SSR or SSE

3.8. Catalysis of NBI to 2CNP with cage and **1.67 mM sulfate** added (0.2 mM NBI, 16.7 mM pH 7 phosphate buffer, 0.125 mM $\mathbf{H}^{W}$). λ_{max} = 410 nm.

Time / s	Abs / a.u.	zero'd	minus bk	[2CNP] / M	[NBI] / M	ln(NBI)
0	0.226	0	0	0.00E+00	2.00E-04	-8.517
120	0.272	0.046	0.04	2.53E-06	1.97E-04	-8.530
240	0.318	0.092	0.082	5.19E-06	1.95E-04	-8.543
360	0.363	0.137	0.121	7.66E-06	1.92E-04	-8.556
480	0.409	0.183	0.16	1.01E-05	1.90E-04	-8.569
600	0.455	0.229	0.202	1.28E-05	1.87E-04	-8.583
720	0.5	0.274	0.24	1.52E-05	1.85E-04	-8.596
840	0.545	0.319	0.279	1.77E-05	1.82E-04	-8.610
960	0.589	0.363	0.316	2.00E-05	1.80E-04	-8.623
1080	0.633	0.407	0.354	2.24E-05	1.78E-04	-8.636
1200	0.675	0.449	0.387	2.45E-05	1.76E-04	-8.648
1320	0.718	0.492	0.424	2.68E-05	1.73E-04	-8.661
1440	0.76	0.534	0.459	2.91E-05	1.71E-04	-8.674
1560	0.801	0.575	0.494	3.13E-05	1.69E-04	-8.687
1680	0.84	0.614	0.525	3.32E-05	1.67E-04	-8.699
1800	0.879	0.653	0.556	3.52E-05	1.65E-04	-8.711
1920	0.916	0.69	0.585	3.70E-05	1.63E-04	-8.722
2040	0.955	0.729	0.617	3.91E-05	1.61E-04	-8.734
2160	0.992	0.766	0.647	4.09E-05	1.59E-04	-8.746

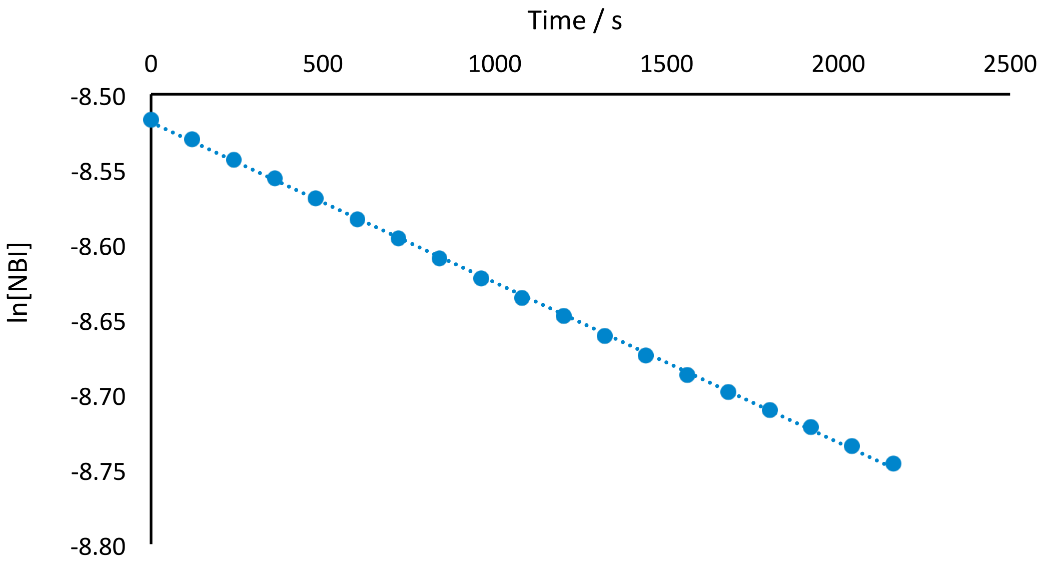

slope	-1.07E-04	-8.52	intercept
SE slope	5.52E-07	6.98E-04	SE intercept
R2	1.000	1.58E-03	SE (y)
F	37343	17	df
SS reg	9.35E-02	4.26E-05	SSR or SSE

3.9. Catalysis of NBI to 2CNP with cage and **1.67 mM iodate** added (0.2 mM NBI, 16.7 mM pH 7 phosphate buffer, 0.125 mM $\mathbf{H}^{\mathbf{W}}$). λ_{max} = 410 nm.

Time / s	Abs / a.u.	zero'd	minus bk	[2CNP] / M	[NBI] / M	ln(NBI)
0	0.236	0	0	0.00E+00	2.00E-04	-8.517
120	0.288	0.052	0.046	2.91E-06	1.97E-04	-8.532
240	0.339	0.103	0.093	5.89E-06	1.94E-04	-8.547
360	0.389	0.153	0.137	8.67E-06	1.91E-04	-8.562
480	0.438	0.202	0.179	1.13E-05	1.89E-04	-8.576
600	0.487	0.251	0.224	1.42E-05	1.86E-04	-8.591
720	0.534	0.298	0.264	1.67E-05	1.83E-04	-8.604
840	0.581	0.345	0.305	1.93E-05	1.81E-04	-8.619
960	0.627	0.391	0.344	2.18E-05	1.78E-04	-8.632
1080	0.673	0.437	0.384	2.43E-05	1.76E-04	-8.647
1200	0.717	0.481	0.419	2.65E-05	1.73E-04	-8.659
1320	0.759	0.523	0.455	2.88E-05	1.71E-04	-8.673
1440	0.8	0.564	0.489	3.09E-05	1.69E-04	-8.685
1560	0.842	0.606	0.525	3.32E-05	1.67E-04	-8.699
1680	0.882	0.646	0.557	3.53E-05	1.65E-04	-8.711
1800	0.921	0.685	0.588	3.72E-05	1.63E-04	-8.723
1920	0.958	0.722	0.617	3.91E-05	1.61E-04	-8.734
2040	0.995	0.759	0.647	4.09E-05	1.59E-04	-8.746
2160	1.03	0.794	0.675	4.27E-05	1.57E-04	-8.757

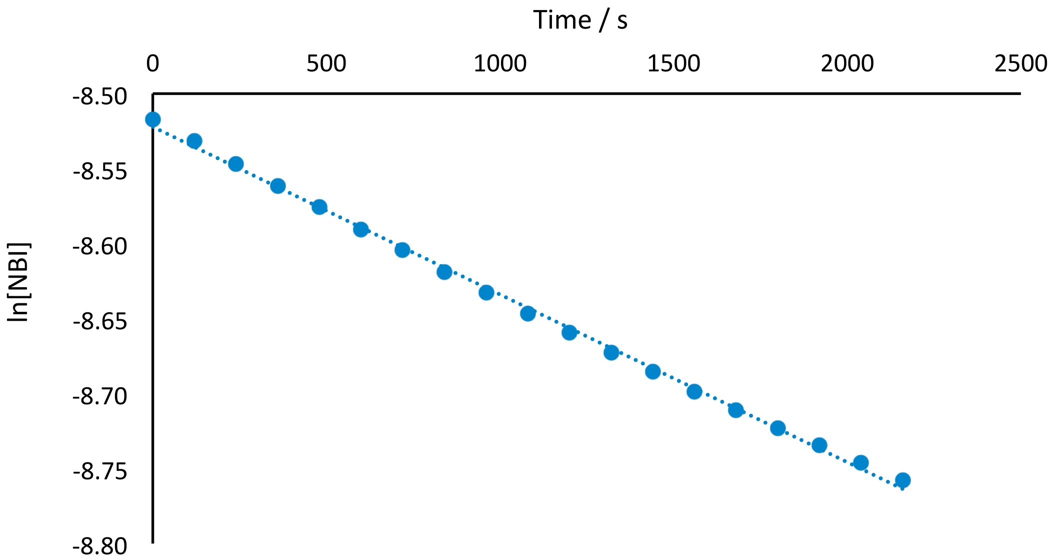

slope	-1.12E-04	-8.52	intercept
SE slope	1.10E-06	1.39E-03	SE intercept
R2	0.998	3.14E-03	SE (y)
F	10396	17	df
SS reg	1.03E-01	1.68E-04	SSR or SSE

3.10. Catalysis of NBI to 2CNP with cage and **1.67 mM bicarbonate** added (0.2 mM NBI, 16.7 mM pH 7 phosphate buffer, 0.125 mM **H^W^**). λ_{max} = 410 nm.

Time / s	Abs / a.u.	zero'd	minus bk	[2CNP] / M	[NBI] / M	ln(NBI)
0	0.429	0	0	0.00E+00	2.00E-04	-8.517
120	0.542	0.113	0.107	6.77E-06	1.93E-04	-8.552
240	0.647	0.218	0.208	1.32E-05	1.87E-04	-8.585
360	0.74	0.311	0.295	1.87E-05	1.81E-04	-8.615
480	0.827	0.398	0.375	2.37E-05	1.76E-04	-8.644
600	0.91	0.481	0.454	2.87E-05	1.71E-04	-8.672
720	0.988	0.559	0.525	3.32E-05	1.67E-04	-8.699
840	1.056	0.627	0.587	3.72E-05	1.63E-04	-8.723
960	1.126	0.697	0.65	4.11E-05	1.59E-04	-8.747
1080	1.188	0.759	0.706	4.47E-05	1.55E-04	-8.770

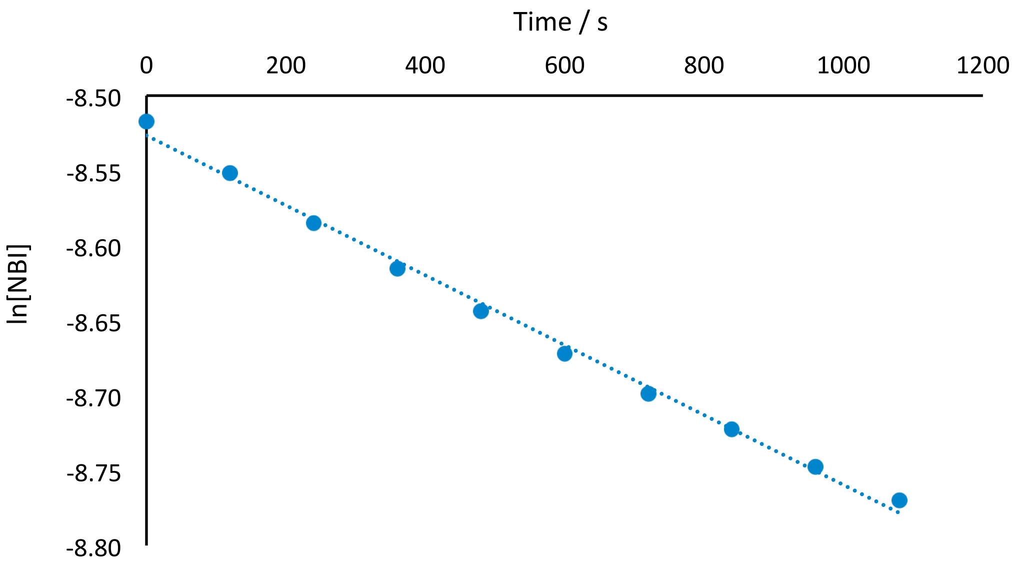

slope	-2.33E-04	-8.53	intercept
SE slope	5.46E-06	3.50E-03	SE intercept
R2	0.996	5.95E-03	SE (y)
F	1823	8	df
SS reg	6.45E-02	2.83E-04	SSR or SSE

3.11. Catalysis of NBI to 2CNP with cage and **16.7 mM fluoride** added (0.2 mM NBI, 16.7 mM pH 7 phosphate buffer, 0.125 mM $\mathbf{H^W}$). λ_{max} = 410 nm.

Time / s	Abs / a.u.	zero'd	minus bk	[2CNP] / M	[NBI] / M	ln(NBI)
0	0.391	0	0	0.00E+00	2.00E-04	-8.517
120	0.482	0.091	0.085	5.38E-06	1.95E-04	-8.544
240	0.57	0.179	0.169	1.07E-05	1.89E-04	-8.572
360	0.654	0.263	0.247	1.56E-05	1.84E-04	-8.599
480	0.735	0.344	0.321	2.03E-05	1.80E-04	-8.624
600	0.813	0.422	0.395	2.50E-05	1.75E-04	-8.651
720	0.892	0.501	0.467	2.96E-05	1.70E-04	-8.677
840	0.965	0.574	0.534	3.38E-05	1.66E-04	-8.702
960	1.037	0.646	0.599	3.79E-05	1.62E-04	-8.727
1080	1.107	0.716	0.663	4.20E-05	1.58E-04	-8.753
1200	1.172	0.781	0.719	4.55E-05	1.54E-04	-8.775
1320	1.238	0.847	0.779	4.93E-05	1.51E-04	-8.800
1440	1.299	0.908	0.833	5.27E-05	1.47E-04	-8.823
1560	1.357	0.966	0.885	5.60E-05	1.44E-04	-8.846
1680	1.411	1.02	0.931	5.89E-05	1.41E-04	-8.866
1800	1.466	1.075	0.978	6.19E-05	1.38E-04	-8.888
1920	1.518	1.127	1.022	6.47E-05	1.35E-04	-8.908
2040	1.568	1.177	1.065	6.74E-05	1.33E-04	-8.928
2160	1.613	1.222	1.103	6.98E-05	1.30E-04	-8.947

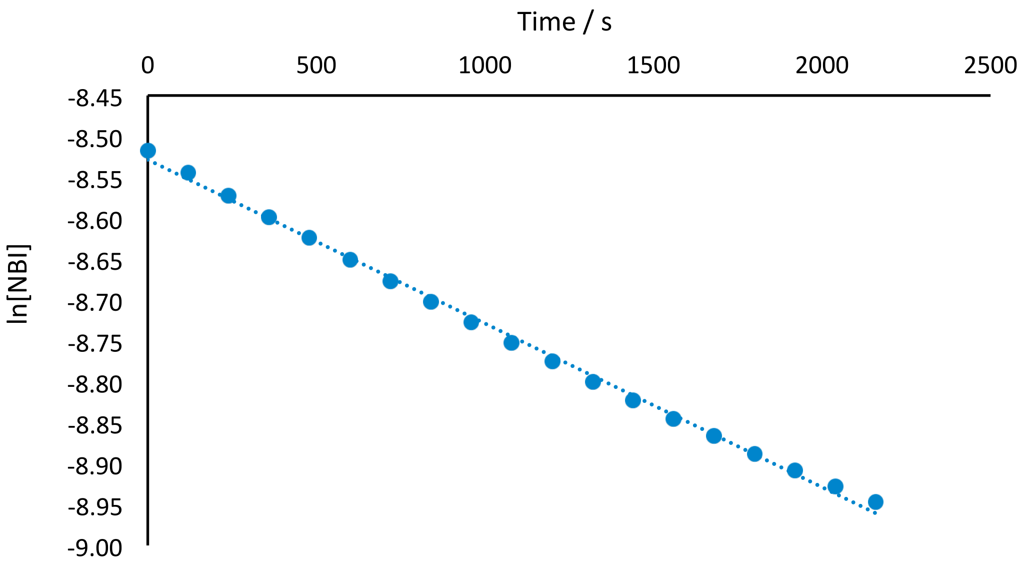

slope	-2.00E-04	-8.53	intercept
SE slope	2.48E-06	3.13E-03	SE intercept
R2	0.997	7.10E-03	SE (y)
F	6519	17	df
SS reg	3.29E-01	8.57E-04	SSR or SSE

3.12. Catalysis of NBI to 2CNP with cage and **16.7 mM chloride** added (0.2 mM NBI, 16.7 mM pH 7 phosphate buffer, 0.125 mM $\mathbf{H}^{\mathbf{W}}$). λ_{max} = 410 nm.

Time / s	Abs / a.u.	zero'd	minus bk	[2CNP] / M	[NBI] / M	ln(NBI)
0	0.187	0	0	0.00E+00	2.00E-04	-8.517
120	0.206	0.019	0.013	8.23E-07	1.99E-04	-8.521
240	0.225	0.038	0.028	1.77E-06	1.98E-04	-8.526
360	0.244	0.057	0.041	2.59E-06	1.97E-04	-8.530
480	0.265	0.078	0.055	3.48E-06	1.97E-04	-8.535
600	0.285	0.098	0.071	4.49E-06	1.96E-04	-8.540
720	0.307	0.12	0.086	5.44E-06	1.95E-04	-8.545
840	0.328	0.141	0.101	6.39E-06	1.94E-04	-8.550
960	0.351	0.164	0.117	7.41E-06	1.93E-04	-8.555
1080	0.374	0.187	0.134	8.48E-06	1.92E-04	-8.561
1200	0.397	0.21	0.148	9.37E-06	1.91E-04	-8.565
1320	0.42	0.233	0.165	1.04E-05	1.90E-04	-8.571
1440	0.443	0.256	0.181	1.15E-05	1.89E-04	-8.576
1560	0.466	0.279	0.198	1.25E-05	1.87E-04	-8.582
1680	0.489	0.302	0.213	1.35E-05	1.87E-04	-8.587
1800	0.512	0.325	0.228	1.44E-05	1.86E-04	-8.592
1920	0.536	0.349	0.244	1.54E-05	1.85E-04	-8.598
2040	0.559	0.372	0.26	1.65E-05	1.84E-04	-8.603
2160	0.583	0.396	0.277	1.75E-05	1.82E-04	-8.609

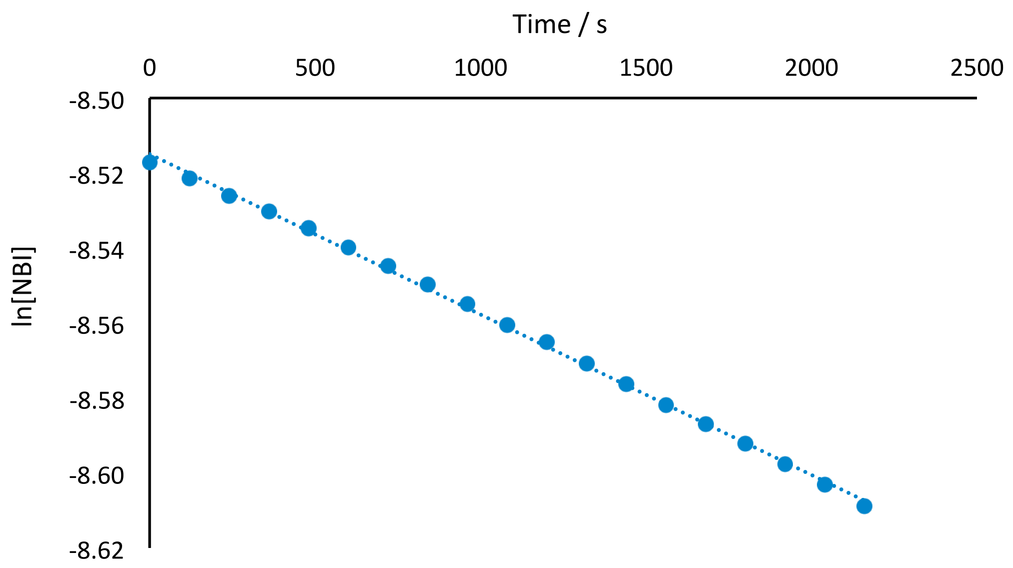

slope	-4.28E-05	-8.51	intercept
SE slope	3.55E-07	4.49E-04	SE intercept
R2	0.999	1.02E-03	SE (y)
F	14513	17	df
SS reg	1.50E-02	1.76E-05	SSR or SSE

3.13. Catalysis of NBI to 2CNP with cage and **16.7 mM bromide** added (0.2 mM NBI, 16.7 mM pH 7 phosphate buffer, 0.125 mM $\mathbf{H^W}$). λ_{max} = 386 nm.

Time / s	Abs / a.u.	zero'd	minus bk	[2CNP] / M	[NBI] / M	ln(NBI)
0	0.356	0	0	0.00E+00	2.00E-04	-8.517
120	0.364	0.008	0.002	1.27E-07	2.00E-04	-8.518
240	0.374	0.018	0.008	5.06E-07	1.99E-04	-8.520
360	0.383	0.027	0.011	6.96E-07	1.99E-04	-8.521
480	0.394	0.038	0.015	9.49E-07	1.99E-04	-8.522
600	0.42	0.064	0.037	2.34E-06	1.98E-04	-8.529
720	0.416	0.06	0.026	1.65E-06	1.98E-04	-8.525
840	0.429	0.073	0.033	2.09E-06	1.98E-04	-8.528
960	0.443	0.087	0.04	2.53E-06	1.97E-04	-8.530
1080	0.454	0.098	0.045	2.85E-06	1.97E-04	-8.532
1200	0.466	0.11	0.048	3.04E-06	1.97E-04	-8.532
1320	0.48	0.124	0.056	3.54E-06	1.96E-04	-8.535
1440	0.494	0.138	0.063	3.99E-06	1.96E-04	-8.537
1560	0.507	0.151	0.07	4.43E-06	1.96E-04	-8.540
1680	0.522	0.166	0.077	4.87E-06	1.95E-04	-8.542
1800	0.536	0.18	0.083	5.25E-06	1.95E-04	-8.544
1920	0.55	0.194	0.089	5.63E-06	1.94E-04	-8.546
2040	0.564	0.208	0.096	6.08E-06	1.94E-04	-8.548
2160	0.58	0.224	0.105	6.65E-06	1.93E-04	-8.551

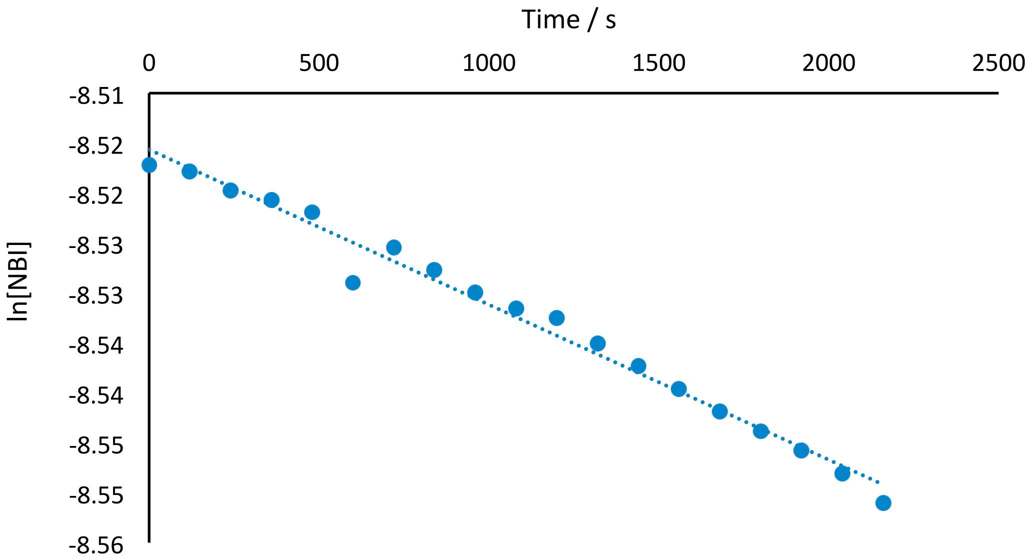

slope	-1.55E-05	-8.52	intercept
SE slope	4.87E-07	6.16E-04	SE intercept
R2	0.984	1.40E-03	SE (y)
F	1016	17	df
SS reg	1.98E-03	3.31E-05	SSR or SSE

3.14. Catalysis of NBI to 2CNP with cage and **16.7 mM nitrate** added (0.2 mM NBI, 16.7 mM pH 7 phosphate buffer, 0.125 mM $\mathbf{H}^{\mathbf{W}}$). λ_{max} = 380 nm.

Time / s	Abs / a.u.	zero'd	minus bk	[2CNP] / M	[NBI] / M	ln(NBI)
0	0.37	0	0	0.00E+00	2.00E-04	-8.517
120	0.38	0.01	0.004	2.53E-07	2.00E-04	-8.518
240	0.39	0.02	0.01	6.33E-07	1.99E-04	-8.520
360	0.398	0.028	0.012	7.59E-07	1.99E-04	-8.521
480	0.408	0.038	0.015	9.49E-07	1.99E-04	-8.522
600	0.419	0.049	0.022	1.39E-06	1.99E-04	-8.524
720	0.429	0.059	0.025	1.58E-06	1.98E-04	-8.525
840	0.44	0.07	0.03	1.90E-06	1.98E-04	-8.527
960	0.454	0.084	0.037	2.34E-06	1.98E-04	-8.529
1080	0.465	0.095	0.042	2.66E-06	1.97E-04	-8.531
1200	0.479	0.109	0.047	2.97E-06	1.97E-04	-8.532
1320	0.492	0.122	0.054	3.42E-06	1.97E-04	-8.534
1440	0.505	0.135	0.06	3.80E-06	1.96E-04	-8.536
1560	0.519	0.149	0.068	4.30E-06	1.96E-04	-8.539
1680	0.533	0.163	0.074	4.68E-06	1.95E-04	-8.541
1800	0.546	0.176	0.079	5.00E-06	1.95E-04	-8.543
1920	0.561	0.191	0.086	5.44E-06	1.95E-04	-8.545
2040	0.575	0.205	0.093	5.89E-06	1.94E-04	-8.547
2160	0.589	0.219	0.1	6.33E-06	1.94E-04	-8.549

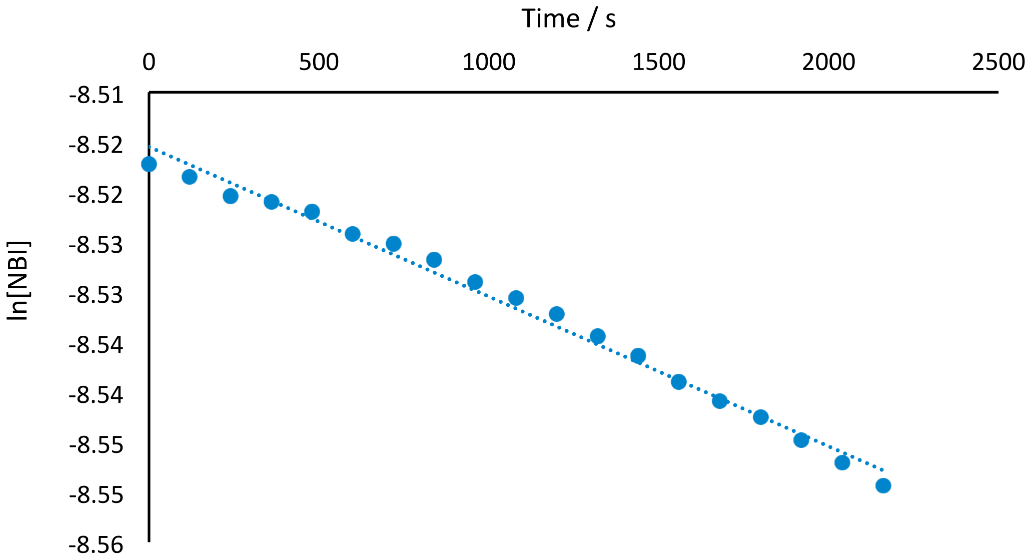

slope	-1.50E-05	-8.52	intercept
SE slope	3.60E-07	4.55E-04	SE intercept
R2	0.990	1.03E-03	SE (y)
F	1728	17	df
SS reg	1.84E-03	1.81E-05	SSR or SSE

3.15. Catalysis of NBI to 2CNP with cage and **16.7 mM sulfate** added (0.2 mM NBI, 16.7 mM pH 7 phosphate buffer, 0.125 mM $\mathbf{H^W}$). λ_{max} = 410 nm.

Time / s	Abs / a.u.	zero'd	minus bk	[2CNP] / M	[NBI] / M	ln(NBI)
0	0.221	0	0	0.00E+00	2.00E-04	-8.517
120	0.258	0.037	0.031	1.96E-06	1.98E-04	-8.527
240	0.294	0.073	0.063	3.99E-06	1.96E-04	-8.537
360	0.331	0.11	0.094	5.95E-06	1.94E-04	-8.547
480	0.37	0.149	0.126	7.97E-06	1.92E-04	-8.558
600	0.409	0.188	0.161	1.02E-05	1.90E-04	-8.569
720	0.447	0.226	0.192	1.22E-05	1.88E-04	-8.580
840	0.487	0.266	0.226	1.43E-05	1.86E-04	-8.591
960	0.526	0.305	0.258	1.63E-05	1.84E-04	-8.602
1080	0.565	0.344	0.291	1.84E-05	1.82E-04	-8.614
1200	0.604	0.383	0.321	2.03E-05	1.80E-04	-8.624
1320	0.644	0.423	0.355	2.25E-05	1.78E-04	-8.636
1440	0.681	0.46	0.385	2.44E-05	1.76E-04	-8.647
1560	0.718	0.497	0.416	2.63E-05	1.74E-04	-8.658
1680	0.755	0.534	0.445	2.82E-05	1.72E-04	-8.669
1800	0.792	0.571	0.474	3.00E-05	1.70E-04	-8.680
1920	0.828	0.607	0.502	3.18E-05	1.68E-04	-8.690
2040	0.863	0.642	0.53	3.35E-05	1.66E-04	-8.701
2160	0.898	0.677	0.558	3.53E-05	1.65E-04	-8.711

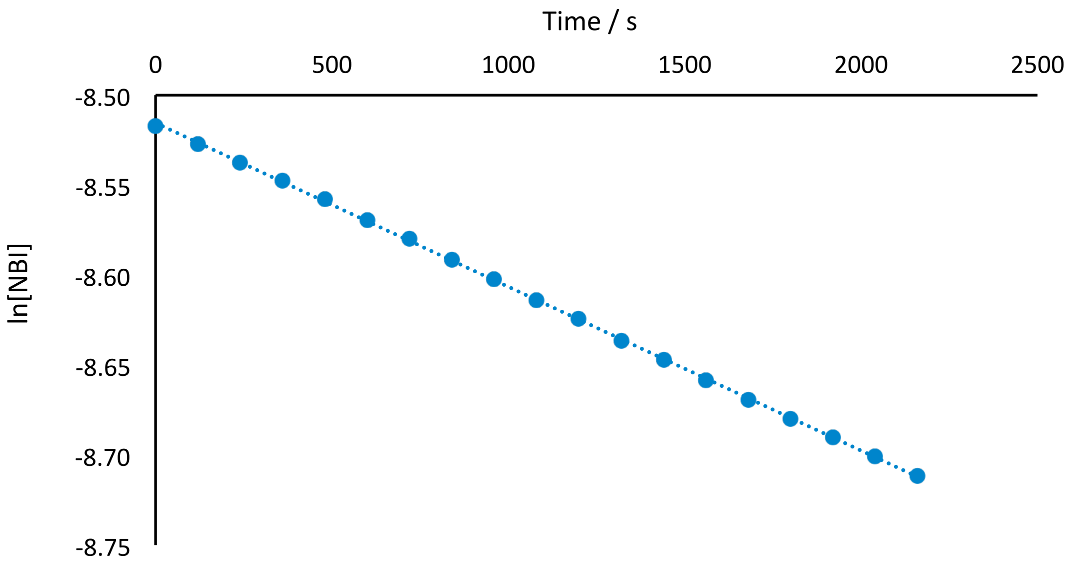

slope	-9.10E-05	-8.52	intercept
SE slope	2.77E-07	3.50E-04	SE intercept
R2	1.000	7.93E-04	SE (y)
F	108243	17	df
SS reg	6.80E-02	1.07E-05	SSR or SSE

3.16. Catalysis of NBI to 2CNP with cage and **16.7 mM iodate** added (0.2 mM NBI, 16.7 mM pH 7 phosphate buffer, 0.125 mM $\mathbf{H^W}$). λ_{max} = 410 nm.

Time / s	Abs / a.u.	zero'd	minus bk	[2CNP] / M	[NBI] / M	ln(NBI)
0	0.251	0	0	0.00E+00	2.00E-04	-8.517
120	0.299	0.048	0.042	2.66E-06	1.97E-04	-8.531
240	0.346	0.095	0.085	5.38E-06	1.95E-04	-8.544
360	0.393	0.142	0.126	7.97E-06	1.92E-04	-8.558
480	0.439	0.188	0.165	1.04E-05	1.90E-04	-8.571
600	0.485	0.234	0.207	1.31E-05	1.87E-04	-8.585
720	0.531	0.28	0.246	1.56E-05	1.84E-04	-8.598
840	0.575	0.324	0.284	1.80E-05	1.82E-04	-8.611
960	0.62	0.369	0.322	2.04E-05	1.80E-04	-8.625
1080	0.664	0.413	0.36	2.28E-05	1.77E-04	-8.638
1200	0.705	0.454	0.392	2.48E-05	1.75E-04	-8.650
1320	0.747	0.496	0.428	2.71E-05	1.73E-04	-8.663
1440	0.788	0.537	0.462	2.92E-05	1.71E-04	-8.675
1560	0.826	0.575	0.494	3.13E-05	1.69E-04	-8.687
1680	0.865	0.614	0.525	3.32E-05	1.67E-04	-8.699
1800	0.903	0.652	0.555	3.51E-05	1.65E-04	-8.710
1920	0.939	0.688	0.583	3.69E-05	1.63E-04	-8.721
2040	0.976	0.725	0.613	3.88E-05	1.61E-04	-8.733
2160	1.01	0.759	0.64	4.05E-05	1.59E-04	-8.744

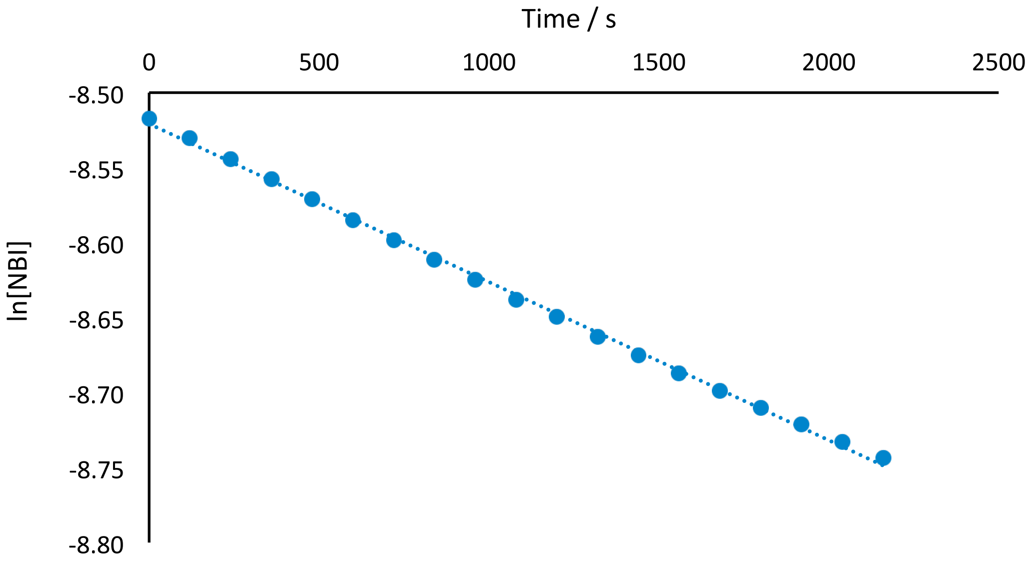

slope	-1.05E-04	-8.52	intercept
SE slope	9.07E-07	1.15E-03	SE intercept
R2	0.999	2.60E-03	SE (y)
F	13537	17	df
SS reg	9.13E-02	1.15E-04	SSR or SSE

4. UV/vis spectroscopic data for varying anion basicity experiments.

4.1. Summary of experiments

All data was obtained as described in Section 2.1.

Sample	Sample name	k_{cat} / s^{-1}	SE k_{cat} / s^{-1}	% error	k_2 / M^{-1} s^{-1}
X1	Background	2.11E-05	2.98E-07	1.4	
X2	H^W	6.91E-05	2.13E-07	0.3	1.38
X3	F^-	7.15E-05	1.58E-07	0.2	1.43
X4	OFm^-	7.64E-05	2.22E-07	0.3	1.53
X5	OAc^-	8.54E-05	3.09E-07	0.4	1.71
X6	HCO_3^-	1.17E-04	1.78E-06	1.5	2.34

4.1. Conversion of NBI to 2CNP under background conditions (0.1 mM NBI, 16.7 mM pH 7 phosphate buffer, no $\mathbf{H^W}$). λ_{max} = 380 nm.

Time / s	Abs / a.u.	Zero'd Abs	[2CNP] / M	[NBI] / M	ln(NBI)
0	0.03	0	0.00E+00	1.00E-04	-9.210
120	0.032	0.002	1.27E-07	9.99E-05	-9.212
240	0.036	0.006	3.80E-07	9.96E-05	-9.214
360	0.039	0.009	5.70E-07	9.94E-05	-9.216
480	0.043	0.013	8.23E-07	9.92E-05	-9.219
600	0.045	0.015	9.49E-07	9.91E-05	-9.220
720	0.05	0.02	1.27E-06	9.87E-05	-9.223
840	0.053	0.023	1.46E-06	9.85E-05	-9.225
960	0.057	0.027	1.71E-06	9.83E-05	-9.228
1080	0.061	0.031	1.96E-06	9.80E-05	-9.230
1200	0.065	0.035	2.22E-06	9.78E-05	-9.233
1320	0.069	0.039	2.47E-06	9.75E-05	-9.235
1440	0.072	0.042	2.66E-06	9.73E-05	-9.237
1560	0.077	0.047	2.97E-06	9.70E-05	-9.241
1680	0.081	0.051	3.23E-06	9.68E-05	-9.243
1800	0.085	0.055	3.48E-06	9.65E-05	-9.246
1920	0.09	0.06	3.80E-06	9.62E-05	-9.249
2040	0.095	0.065	4.11E-06	9.59E-05	-9.252
2160	0.098	0.068	4.30E-06	9.57E-05	-9.254
2280	0.103	0.073	4.62E-06	9.54E-05	-9.258

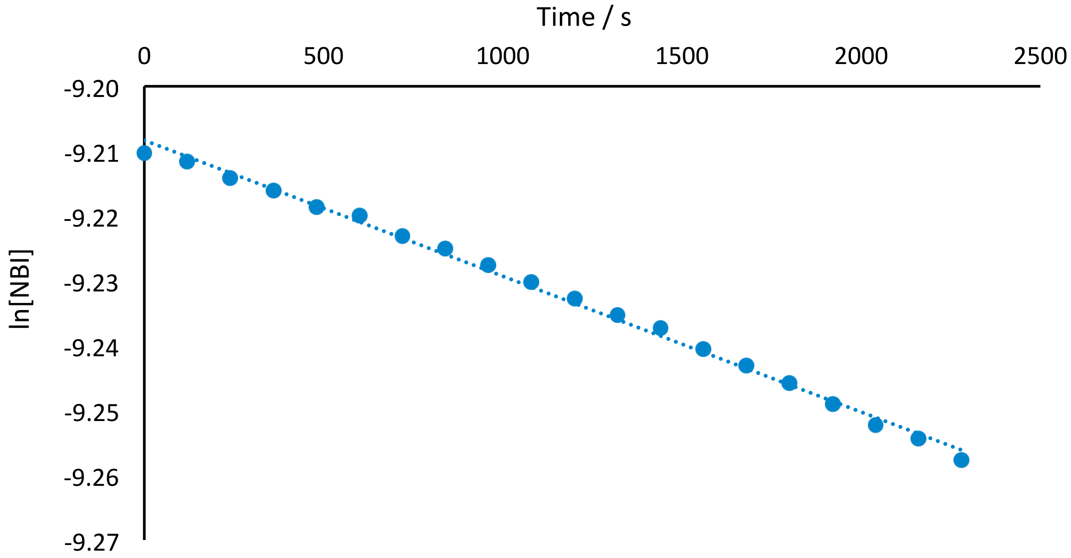

slope	-2.11E-05	-9.21	intercept
SE slope	2.98E-07	4.18E-04	SE intercept
R2	0.996	9.92E-04	SE (y)
F	5032	19	df
SS reg	4.95E-03	1.87E-05	SSR or SSE

4.2. Catalysis of NBI to 2CNP with **0.05 mM cage** added (0.1 mM NBI, 16.7 mM pH 7 phosphate buffer, 0.05 mM $\mathbf{H}^{\mathbf{W}}$). λ_{max} = 406 nm.

Time / s	Abs / a.u.	Zero'd Abs	corrected	[2CNP] / M	[NBI] / M	ln(NBI)
0	0.111	0	0	0.00E+00	1.00E-04	-9.210
120	0.126	0.015	0.013	8.23E-07	9.92E-05	-9.219
240	0.141	0.03	0.024	1.52E-06	9.85E-05	-9.226
360	0.156	0.045	0.036	2.28E-06	9.77E-05	-9.233
480	0.173	0.062	0.049	3.10E-06	9.69E-05	-9.242
600	0.188	0.077	0.062	3.92E-06	9.61E-05	-9.250
720	0.204	0.093	0.073	4.62E-06	9.54E-05	-9.258
840	0.221	0.11	0.087	5.51E-06	9.45E-05	-9.267
960	0.237	0.126	0.099	6.27E-06	9.37E-05	-9.275
1080	0.254	0.143	0.112	7.09E-06	9.29E-05	-9.284
1200	0.27	0.159	0.124	7.85E-06	9.22E-05	-9.292
1320	0.286	0.175	0.136	8.61E-06	9.14E-05	-9.300
1440	0.302	0.191	0.149	9.43E-06	9.06E-05	-9.309
1560	0.318	0.207	0.16	1.01E-05	8.99E-05	-9.317
1680	0.335	0.224	0.173	1.09E-05	8.91E-05	-9.326
1800	0.351	0.24	0.185	1.17E-05	8.83E-05	-9.335
1920	0.366	0.255	0.195	1.23E-05	8.77E-05	-9.342
2040	0.382	0.271	0.206	1.30E-05	8.70E-05	-9.350
2160	0.397	0.286	0.218	1.38E-05	8.62E-05	-9.359
2280	0.412	0.301	0.228	1.44E-05	8.56E-05	-9.366

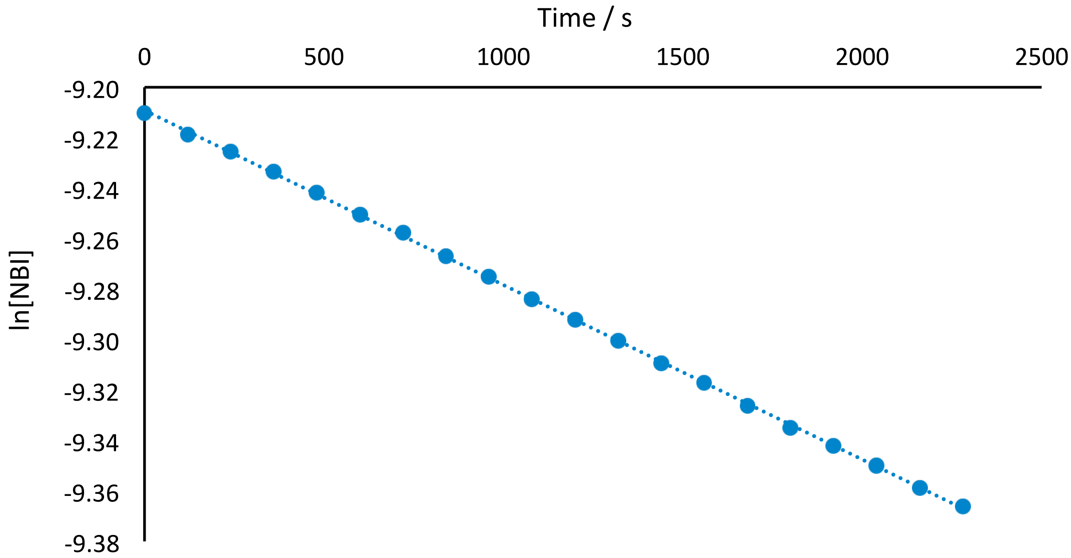

slope	-6.91E-05	-9.21	intercept
SE slope	2.13E-07	2.99E-04	SE intercept
R2	1.000	7.09E-04	SE (y)
F	105348	19	df
SS reg	5.29E-02	9.55E-06	SSR or SSE

4.3. Catalysis of NBI to 2CNP with **1 mM fluoride** added (0.1 mM NBI, 16.7 mM pH 7 phosphate buffer, 0.05 mM $\mathbf{H}^{\mathbf{W}}$). λ_{max} = 406 nm.

Time / s	Abs / a.u.	Zero'd Abs	corrected	[2CNP] / M	[NBI] / M	ln(NBI)
0	0.115	0	0	0.00E+00	1.00E-04	-9.210
120	0.131	0.016	0.014	8.86E-07	9.91E-05	-9.219
240	0.147	0.032	0.026	1.65E-06	9.84E-05	-9.227
360	0.164	0.049	0.04	2.53E-06	9.75E-05	-9.236
480	0.18	0.065	0.052	3.29E-06	9.67E-05	-9.244
600	0.196	0.081	0.066	4.18E-06	9.58E-05	-9.253
720	0.214	0.099	0.079	5.00E-06	9.50E-05	-9.262
840	0.23	0.115	0.092	5.82E-06	9.42E-05	-9.270
960	0.246	0.131	0.104	6.58E-06	9.34E-05	-9.278
1080	0.264	0.149	0.118	7.47E-06	9.25E-05	-9.288
1200	0.28	0.165	0.13	8.23E-06	9.18E-05	-9.296
1320	0.297	0.182	0.143	9.05E-06	9.09E-05	-9.305
1440	0.312	0.197	0.155	9.81E-06	9.02E-05	-9.314
1560	0.329	0.214	0.167	1.06E-05	8.94E-05	-9.322
1680	0.346	0.231	0.18	1.14E-05	8.86E-05	-9.331
1800	0.362	0.247	0.192	1.22E-05	8.78E-05	-9.340
1920	0.378	0.263	0.203	1.28E-05	8.72E-05	-9.348
2040	0.394	0.279	0.214	1.35E-05	8.65E-05	-9.356
2160	0.408	0.293	0.225	1.42E-05	8.58E-05	-9.364
2280	0.425	0.31	0.237	1.50E-05	8.50E-05	-9.373

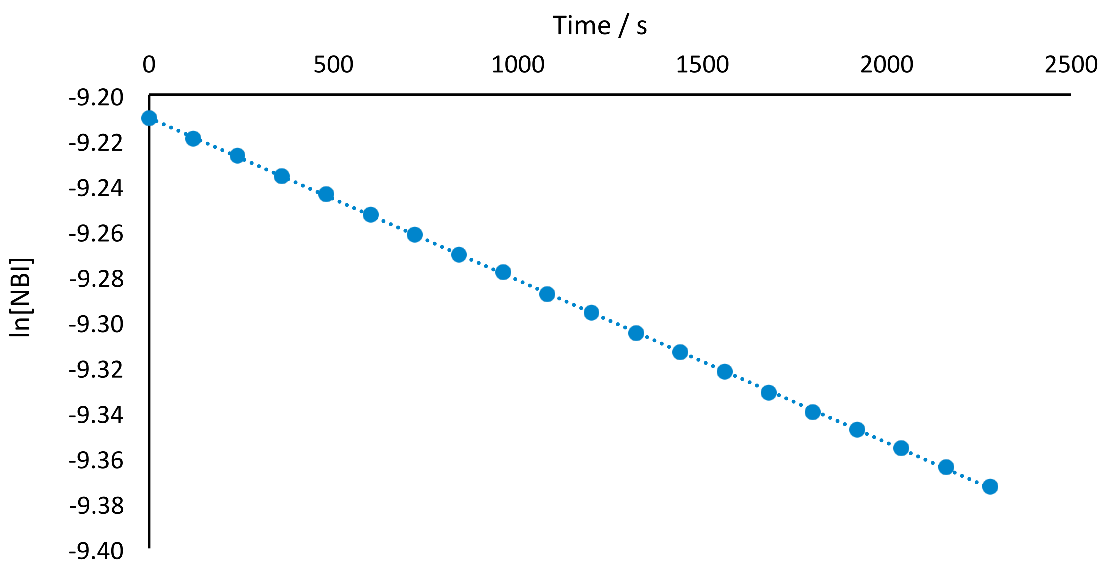

slope	-7.15E-05	-9.21	intercept
SE slope	1.58E-07	2.21E-04	SE intercept
R2	1.000	5.25E-04	SE (y)
F	205886	19	df
SS reg	5.67E-02	5.23E-06	SSR or SSE

4.4. Catalysis of NBI to 2CNP with **1 mM formate** added (0.1 mM NBI, 16.7 mM pH 7 phosphate buffer, 0.05 mM $\mathbf{H^W}$). λ_{max} = 406 nm.

Time / s	Abs / a.u.	Zero'd Abs	corrected	[2CNP] / M	[NBI] / M	ln(NBI)
0	0.109	0	0	0.00E+00	1.00E-04	-9.210
120	0.126	0.017	0.015	9.49E-07	9.91E-05	-9.220
240	0.143	0.034	0.028	1.77E-06	9.82E-05	-9.228
360	0.16	0.051	0.042	2.66E-06	9.73E-05	-9.237
480	0.178	0.069	0.056	3.54E-06	9.65E-05	-9.246
600	0.194	0.085	0.07	4.43E-06	9.56E-05	-9.256
720	0.212	0.103	0.083	5.25E-06	9.47E-05	-9.264
840	0.23	0.121	0.098	6.20E-06	9.38E-05	-9.274
960	0.246	0.137	0.11	6.96E-06	9.30E-05	-9.283
1080	0.265	0.156	0.125	7.91E-06	9.21E-05	-9.293
1200	0.283	0.174	0.139	8.80E-06	9.12E-05	-9.302
1320	0.3	0.191	0.152	9.62E-06	9.04E-05	-9.311
1440	0.317	0.208	0.166	1.05E-05	8.95E-05	-9.321
1560	0.334	0.225	0.178	1.13E-05	8.87E-05	-9.330
1680	0.352	0.243	0.192	1.22E-05	8.78E-05	-9.340
1800	0.368	0.259	0.204	1.29E-05	8.71E-05	-9.349
1920	0.385	0.276	0.216	1.37E-05	8.63E-05	-9.357
2040	0.401	0.292	0.227	1.44E-05	8.56E-05	-9.365
2160	0.417	0.308	0.24	1.52E-05	8.48E-05	-9.375
2280	0.433	0.324	0.251	1.59E-05	8.41E-05	-9.383

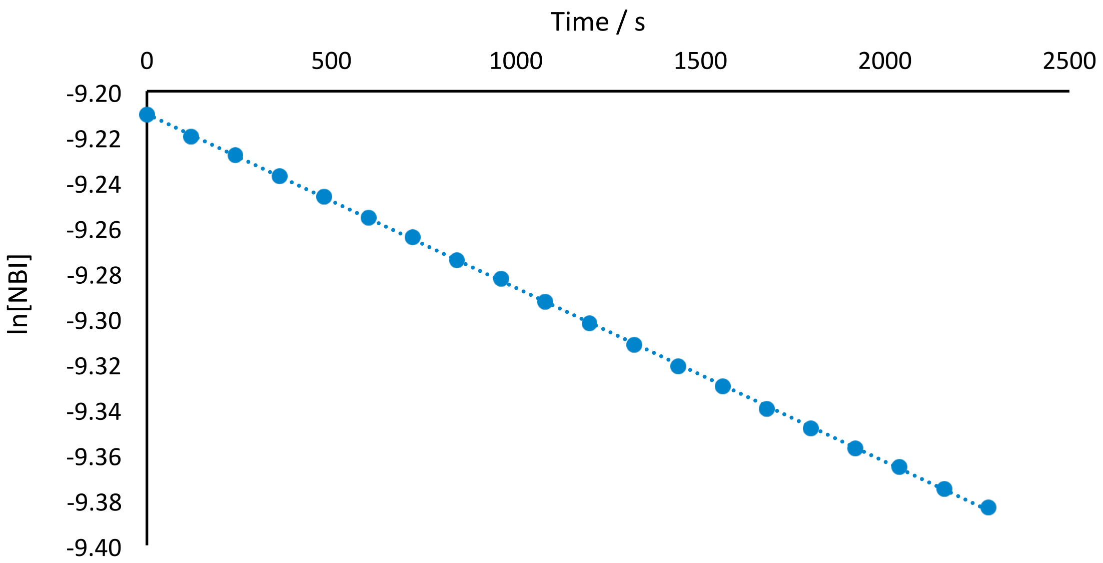

slope	-7.64E-05	-9.21	intercept
SE slope	2.22E-07	3.12E-04	SE intercept
R2	1.000	7.40E-04	SE (y)
F	118211.954	19	df
SS reg	6.47E-02	1.04E-05	SSR or SSE

4.5. Catalysis of NBI to 2CNP with **1 mM acetate** added (0.1 mM NBI, 16.7 mM pH 7 phosphate buffer, 0.05 mM $\mathbf{H}^{\mathbf{W}}$). λ_{max} = 406 nm.

Time / s	Abs / a.u.	Zero'd Abs	corrected	[2CNP] / M	[NBI] / M	ln(NBI)
0	0.112	0	0	0.00E+00	1.00E-04	-9.210
120	0.13	0.018	0.016	1.01E-06	9.90E-05	-9.221
240	0.149	0.037	0.031	1.96E-06	9.80E-05	-9.230
360	0.168	0.056	0.047	2.97E-06	9.70E-05	-9.241
480	0.186	0.074	0.061	3.86E-06	9.61E-05	-9.250
600	0.204	0.092	0.077	4.87E-06	9.51E-05	-9.260
720	0.224	0.112	0.092	5.82E-06	9.42E-05	-9.270
840	0.244	0.132	0.109	6.90E-06	9.31E-05	-9.282
960	0.262	0.15	0.123	7.78E-06	9.22E-05	-9.291
1080	0.282	0.17	0.139	8.80E-06	9.12E-05	-9.302
1200	0.301	0.189	0.154	9.75E-06	9.03E-05	-9.313
1320	0.32	0.208	0.169	1.07E-05	8.93E-05	-9.323
1440	0.339	0.227	0.185	1.17E-05	8.83E-05	-9.335
1560	0.357	0.245	0.198	1.25E-05	8.75E-05	-9.344
1680	0.376	0.264	0.213	1.35E-05	8.65E-05	-9.355
1800	0.393	0.281	0.226	1.43E-05	8.57E-05	-9.365
1920	0.411	0.299	0.239	1.51E-05	8.49E-05	-9.374
2040	0.429	0.317	0.252	1.59E-05	8.41E-05	-9.384
2160	0.445	0.333	0.265	1.68E-05	8.32E-05	-9.394
2280	0.462	0.35	0.277	1.75E-05	8.25E-05	-9.403

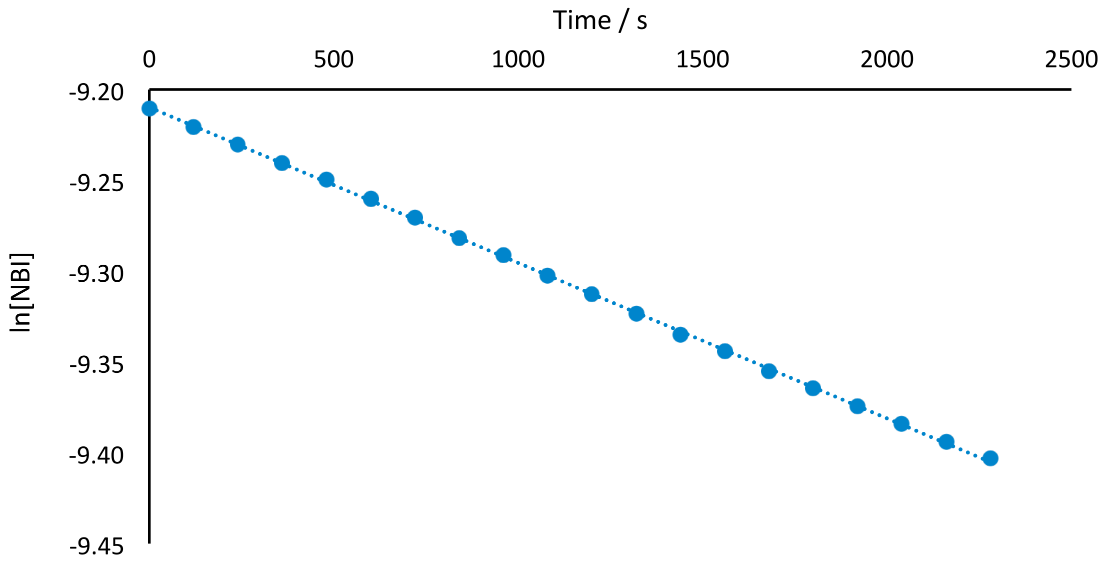

slope	-8.54E-05	-9.21	intercept
SE slope	3.09E-07	4.33E-04	SE intercept
R2	1.000	1.03E-03	SE (y)
F	76394	19	df
SS reg	8.09E-02	2.01E-05	SSR or SSE

4.6. Catalysis of NBI to 2CNP with **1 mM bicarbonate** added (0.1 mM NBI, 16.7 mM pH 7 phosphate buffer, 0.05 mM $\mathbf{H}^{\mathbf{W}}$). λ_{max} = 406 nm.

Time / s	Abs / a.u.	Zero'd Abs	corrected	[2CNP] / M	[NBI] / M	ln(NBI)
0	0.127	0	0	0.00E+00	1.00E-04	-9.210
120	0.157	0.03	0.028	1.77E-06	9.82E-05	-9.228
240	0.186	0.059	0.053	3.35E-06	9.66E-05	-9.244
360	0.215	0.088	0.079	5.00E-06	9.50E-05	-9.262
480	0.243	0.116	0.103	6.52E-06	9.35E-05	-9.278
600	0.27	0.143	0.128	8.10E-06	9.19E-05	-9.295
720	0.296	0.169	0.149	9.43E-06	9.06E-05	-9.309
840	0.321	0.194	0.171	1.08E-05	8.92E-05	-9.325
960	0.345	0.218	0.191	1.21E-05	8.79E-05	-9.339
1080	0.37	0.243	0.212	1.34E-05	8.66E-05	-9.354
1200	0.393	0.266	0.231	1.46E-05	8.54E-05	-9.368
1320	0.415	0.288	0.249	1.58E-05	8.42E-05	-9.382
1440	0.437	0.31	0.268	1.70E-05	8.30E-05	-9.396
1560	0.458	0.331	0.284	1.80E-05	8.20E-05	-9.408
1680	0.479	0.352	0.301	1.91E-05	8.09E-05	-9.422
1800	0.499	0.372	0.317	2.01E-05	7.99E-05	-9.434
1920	0.519	0.392	0.332	2.10E-05	7.90E-05	-9.446
2040	0.537	0.41	0.345	2.18E-05	7.82E-05	-9.457
2160	0.555	0.428	0.36	2.28E-05	7.72E-05	-9.469
2280	0.574	0.447	0.374	2.37E-05	7.63E-05	-9.480

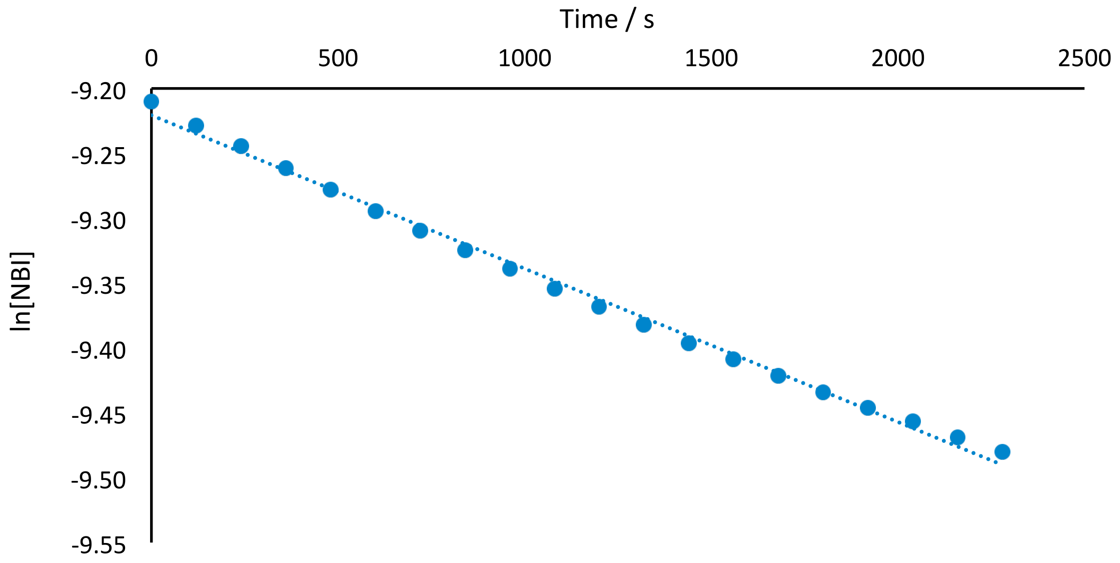

slope	-1.17E-04	-9.22	intercept
SE slope	1.78E-06	2.50E-03	SE intercept
R2	0.996	5.94E-03	SE (y)
F	4295	19	df
SS reg	1.52E-01	6.71E-04	SSR or SSE

5. Additional figures

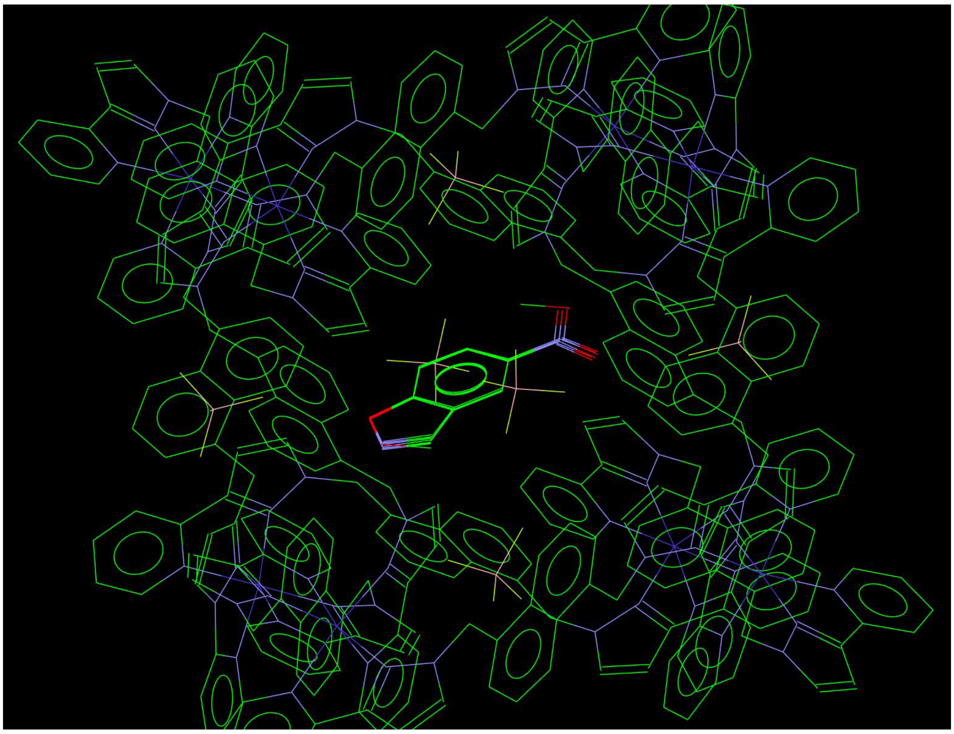

Figure S2. Energy-minimised structure of the cage/NBI 1:1 complex obtained using GOLD (see main text). The NBI was posed initially in four different orientations and all converged to the same minimum-energy structure (see four overlays).

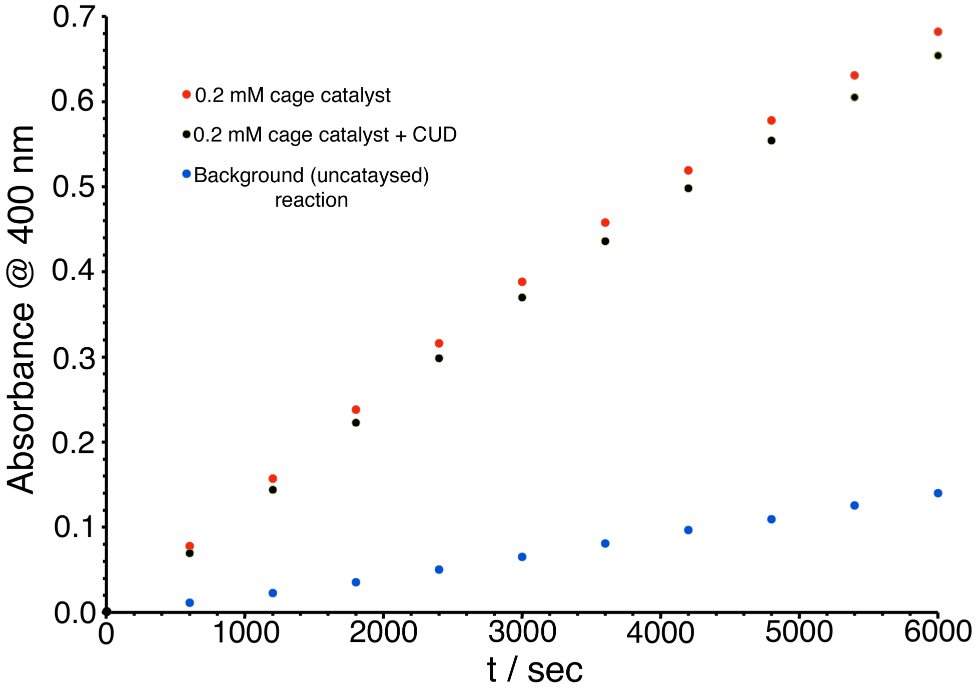

Figure S3. Rate of cage-catalysed reaction (Kemp elimination with NBI) using cage alone (red data points) and then after addition of sufficient cyclundecanone to block the cage cavity (black data points) – showing virtually no change from blocking the cavity. For reference the rate of the uncatalysed background reaction is shown with blue data points.

References

S1 M. Whitehead, S. Turega, A. Stephenson, C. A. Hunter, and M. D. Ward, *Chem. Sci.,* 2013, **4**, 2744.

S2 I. V. Korendovych, D. W. Kulp, Y. Wu, H. Cheng, H. Roder and W. F. DeGrado, *Proc. Natl. Acad. Sci. U. S. A.*, 2011, **108**, 6823–6827.
